# “*Nano Lab*” Advanced Characterization Platform for Studying Electrocatalytic Iridium Nanoparticles Dispersed on TiO_*x*_N_*y*_ Supports Prepared on Ti Transmission Electron Microscopy Grids

**DOI:** 10.1021/acsanm.3c01368

**Published:** 2023-06-05

**Authors:** Marjan Bele, Gorazd Koderman Podboršek, Anja Lončar, Primož Jovanovič, Armin Hrnjić, Živa Marinko, Janez Kovač, Angelja Kjara Surca, Ana Rebeka Kamšek, Goran Dražić, Nejc Hodnik, Luka Suhadolnik

**Affiliations:** † Department of Materials Chemistry, National Institute of Chemistry, Hajdrihova 19, Ljubljana SI-1000, Slovenia; ‡ Jožef Stefan International Postgraduate School, Jamova 39, Ljubljana SI-1000, Slovenia; § University of Nova Gorica, Vipavska 13, Nova Gorica SI-5000, Slovenia; ∥ Department for Nanostructured Materials, Jožef Stefan Institute, Jamova 39, Ljubljana SI-1000, Slovenia; ⊥ Department of Surface Engineering, Jožef Stefan Institute, Jamova 39, Ljubljana SI-1000, Slovenia; # Faculty of Chemistry and Chemical Technology, University of Ljubljana, Večna pot 113, Ljubljana SI-1000, Slovenia; ¶ Department of Chemical and Pharmaceutical Sciences, University of Trieste, via L. Giorgieri 1, Trieste 34127, Italy

**Keywords:** *Nano Lab* concept, anodic oxidation, IL-TEM, electrocatalysis, oxygen evolution reaction, iridium nanoparticles

## Abstract

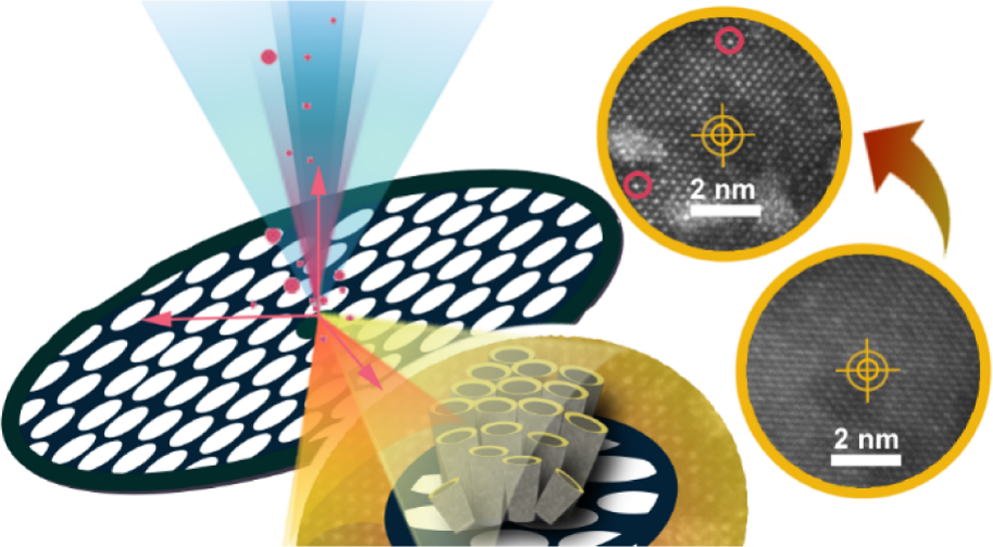

Aiming at speeding up the discovery and understanding of promising electrocatalysts, a novel experimental platform, *i.e.*, the *Nano Lab*, is introduced. It is based on state-of-the-art physicochemical characterization and atomic-scale tracking of individual synthesis steps as well as subsequent electrochemical treatments targeting nanostructured composites. This is provided by having the entire experimental setup on a transmission electron microscopy (TEM) grid. Herein, the oxygen evolution reaction nanocomposite electrocatalyst, i.e., iridium nanoparticles dispersed on a high-surface-area TiO_*x*_N_*y*_ support prepared on the Ti TEM grid, is investigated. By combining electrochemical concepts such as anodic oxidation of TEM grids, floating electrode-based electrochemical characterization, and identical location TEM analysis, relevant information from the entire composite’s cycle, *i.e.*, from the initial synthesis step to electrochemical operation, can be studied. We reveal that Ir nanoparticles as well as the TiO_*x*_N_*y*_ support undergo dynamic changes during all steps. The most interesting findings made possible by the *Nano Lab* concept are the formation of Ir single atoms and only a small decrease in the N/O ratio of the TiO_*x*_N_*y*_–Ir catalyst during the electrochemical treatment. In this way, we show that the precise influence of the nanoscale structure, composition, morphology, and electrocatalyst’s locally resolved surface sites can be deciphered on the atomic level. Furthermore, the *Nano Lab*’s experimental setup is compatible with *ex situ* characterization and other analytical methods, such as Raman spectroscopy, X-ray photoelectron spectroscopy, and identical location scanning electron microscopy, hence providing a comprehensive understanding of structural changes and their effects. Overall, an experimental toolbox for the systematic development of supported electrocatalysts is now at hand.

## Introduction

Electrochemical conversion of renewable resources to electrical energy, fuels, or useful chemicals is one of the most promising directions for humankind to meet the most urgent technological goals, namely, a clean energy landscape. Herein, nanostructured electrocatalysts will play the central role, meaning that their timely development is of utmost importance. Unfortunately, widespread philosophy is still strongly based on a trial-and-error approach, where a plethora of synthesis strategies and the corresponding structural motifs have been reported. Even though these electrocatalysts demonstrate relevant catalytic performances, it is very challenging to interpret the observed electrochemical results and identify the modifications to active sites imposed by electrochemical testing. Accordingly, future perspectives should be directed toward obtaining insights on the nano-to-atomic scale, where the development of advanced experimental techniques capable of local phenomenon isolation is paramount. Coupling electrochemical techniques and structural analysis, in particular, would be highly suitable. From a nanostructural perspective, electron microscopy is the method of choice as it can resolve surface and near-surface structures. It can be divided into three categories, namely, *ex situ*, *quasi in situ*, and *in situ*.^1^ In the former case, the analysis of the observed nanoscale phenomenon can only be statistically relevant when observed on a large volume of nanostructures. In the latter case, many technical challenges are still unresolved, for instance, the strong interaction of the electron beam with the supporting electrolyte and the electrode material, to allow for reproducible measurements without artifacts and fair interpretation. Therefore, in practice, both approaches are very problematic for studying nanostructures that are very diverse. While the *in situ* approach is the ultimate solution, the *ex situ* approach suffers from limitations originating from the complexity of the studied systems. For instance, electrocatalysts consist of billions of nanoparticles, with each having many unique nanofeatures like a sequence of defects, which make their changes impossible to track. To circumvent this issue, the so-called identical location electron microscopy approach, a *quasi in situ* method introduced by Mayrhofer in 2008^2^ and further developed by our group, is much more meaningful. It presents a link to understanding the mechanism of catalyst degradation as it enables tracking and thus resolving the changes of the structures of the nanoparticles down to the atomic level.^3^ In pursuit of this philosophy, we introduce herein a novel experimental platform referred to as the *Nano Lab.* The platform allows observation of nanoscale events of the entire catalyst’s life cycle, *i.e.*, synthesis as well as electrochemical biasing. An important addition to that is the reliable electrochemical characterization ensured by the *Nano Lab*. Note that this is not trivial in typical identical location-transmission electron microscopy (IL-TEM) experiments, which use TEM grids with a thin layer of a specimen to ensure electron beam transparency. However, the grids themselves are extremely delicate and prone to damage; hence, it is challenging to handle them multiple times. This is even more pronounced while being used repeatedly in electrochemical experiments.^4^ In addition to that, TEM grids are usually pressed onto the glassy carbon rotating disc electrode (RDE) with a specially designed cup.^2,5−8^ This approach does not allow for a well-resolved electrochemical signal as the transport of the reactant is hindered by the cap, the altered hydrodynamics of the RDE, and the extremely low amounts of deposited catalyst. Instead, an adequate electrochemical setup with a modified floating-type electrode configuration, originally developed by Kucernak’s group,^9−12^ was recently adopted by our group.^13−15^ In this case, the TEM grid can serve as the working electrode (WE) which is placed on the surface of the electrolyte solution, unlike conventional TEM grid-based electrodes which are fully immersed in the electrolyte.^16^ This particular configuration enables the electrochemical reaction to proceed at well-resolved and elevated currents, allowing for a direct correlation between the electrocatalytic performance and structural changes. Importantly, tracking of atomically resolved structural and compositional changes of a catalyst at an identical location *before* and *after* consecutive electrochemical treatments *via* TEM analysis is possible.^13,17,18^ In the present study, these capabilities were substantially upgraded. More specifically:

(i) The drawbacks of wet drop casting for catalyst deposition on the TEM, leading to inhomogeneous deposits and an unstable catalyst layer, are circumvented. This is achieved by using the TEM grid as a specimen itself. For instance, the anodization of a Ti TEM grid with subsequent annealing and nitridation leads to conductive high-surface-area electrodes. These are also sufficiently stable to perform long-term electrochemical experiments under harsh conditions. We demonstrate this by immobilizing iridium nanoparticles and performing subsequent oxygen evolution reaction (OER) characterization.
(ii) As anodically oxidized TEM grid already presents a relevant high-surface-area OER support, individual synthesis steps can be inspected with IL-TEM methodology.
(iii) TEM grids can be characterized with other non-destructive analyses like X-ray photoelectron spectroscopy (XPS), Raman spectroscopy, and scanning electron microscopy (SEM), which offer unique complementary information about the catalyst.

The effectiveness of such *Nano Lab* is herein demonstrated on a relevant OER composite, *i.e.*, iridium supported on titanium oxynitride (TiO_*x*_N_*y*_–Ir). We demonstrate that the *Nano Lab* platform allows for advanced observation and tracking of how an electrocatalyst’s local surface, morphology, structure, and composition change at a micrometer and down to the atomic level. Through careful synthesis and comprehensive characterization, we reveal that Ir nanoparticles, as well as TiO_*x*_N_*y*_ supports, undergo dynamic changes during all steps.

## Experimental Section

### Synthesis of the TiON–Ir Catalyst

**Figure 1:**
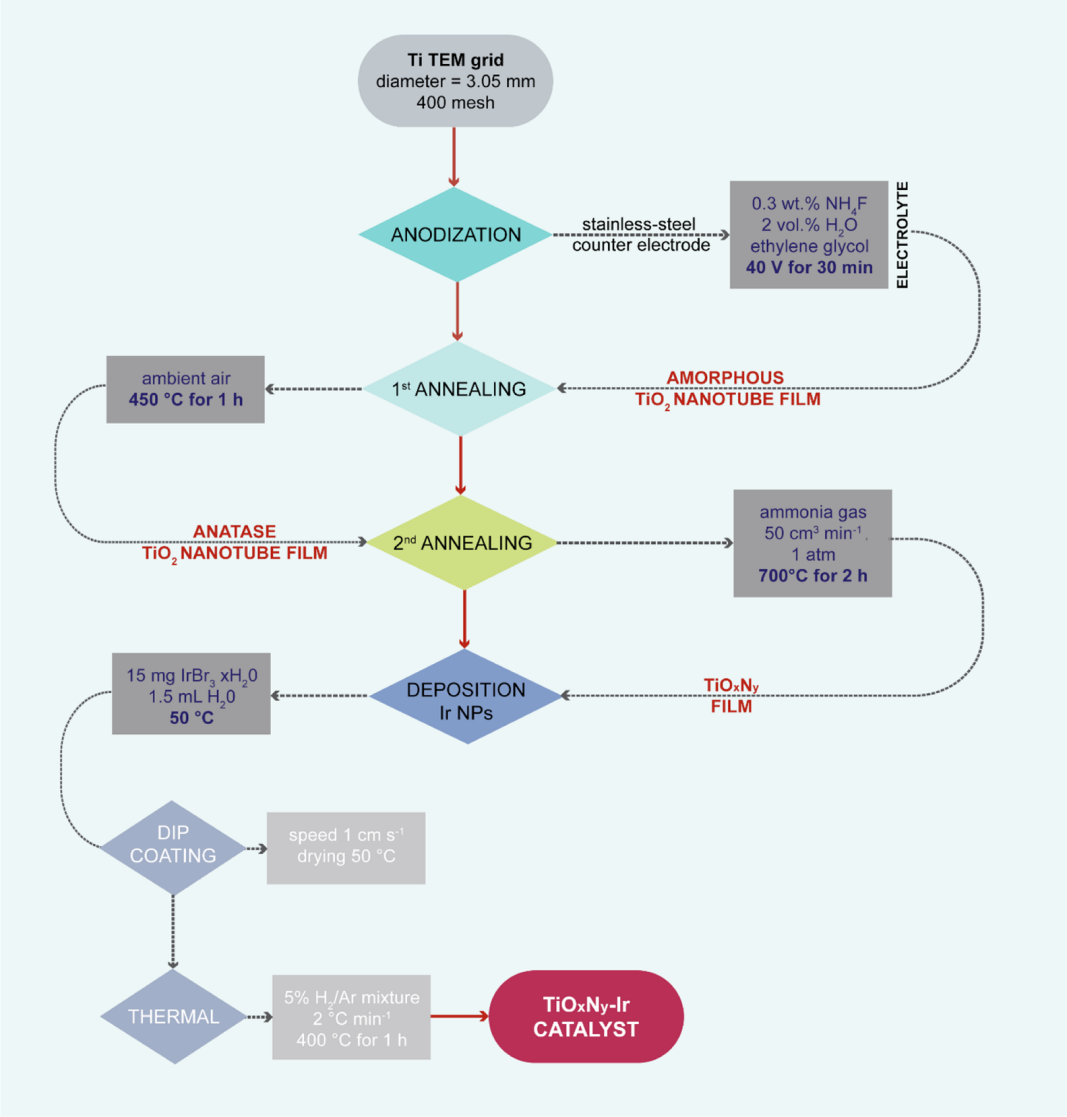
Flowchart of the TiO_*x*_N_*y*_–Ir floating electrode catalyst synthesis, including all major synthesis parameters.

The TiO_*x*_N_*y*_–Ir catalyst was synthesized in a stepwise protocol as described in detail in our recent publication (Figure 1).^19^ Briefly, initially, the Ti TEM grid (3.05 mm diameter, 400 mesh, SPI Supplies) underwent potentiostatic anodization in a two-electrode cell using a stainless-steel counter electrode at a constant voltage of 40 V for 30 min, resulting in an amorphous TiO_2_ nanotube film. For this purpose, a recently developed anodization apparatus was employed.^20^ After subsequent two-step annealing in air and ammonia atmosphere, respectively, a crystalline TiO_*x*_N_*y*_ substrate was obtained. In the final step, iridium nanoparticles were deposited from the iridium (III) bromide precursor *via* dip-coating and subsequently annealed in a reductive atmosphere (5% H_2_/Ar mixture, 400 °C).

### Materials Characterization

#### Optical Microscopy

Optical microscopy was done using Optika Microscopes SZM-B (Ponteranica, Italy).

#### Scanning Electron Microscopy

Identical location-SEM (IL-SEM) was carried out using a Zeiss Supra TM 35 VP (Carl Zeiss, Oberkochen, Germany) field emission scanning electron microscope. The sample TEM grids were put on a scanning transmission electron microscopy (STEM) holder. The operational voltage was set to 1 kV, working distance was 4.9 mm, aperture size was 30 μm, and the detectors were a secondary electron detector and a back-scattered electron detector.

#### Scanning Transmission Electron Microscopy

Cs-corrected transmission electron microscopy (CF-ARM JEOL 200) was utilized for IL-STEM imaging, incorporating an SSD JEOL energy-dispersive X-ray (EDX) spectrometer and a GATAN Quantum ER dual-electron energy loss spectrometer. An operational voltage of 80 kV was employed. The imaging was conducted in the STEM mode, capturing both bright-field (BF) and high-angle annular dark-field (HAADF) images with a probe size of 6 C and an effective camera length of 8 cm. Electron energy loss spectroscopy (EELS) analysis was performed using a probe size of 6 C and an effective camera length of 3 cm, while EDX spectroscopy analysis utilized a probe size of 2 C and an effective camera length of 8 cm.

#### Statistical Analysis of Ir Nanoparticles

An in-house algorithm based on adaptive thresholding was employed for nanoparticle segmentation. Geometric parameters were collected with Fiji (ImageJ).^21^

#### Raman Spectroscopy

Raman spectra of the TEM grid samples were obtained using a confocal WITec alpha 300 Raman spectrometer. The spectra were recorded with a 532 nm laser excitation light and an integration time of 2 s after each preparation step (*i.e.*, air-annealed, nitridated, and TiO_*x*_N_*y*_–Ir samples) and finally after EC-STAT analysis. The air-annealed form of the sample was extremely sensitive to laser light excitation, demanding a low laser power of 0.6 mW and also a diminished number of scans [from 100 (a) to 20 (b,c) scans in Figure S1]. These spectra are background corrected (due to fluorescence), while spectra of others are shown, as measured in insets in Figure S2. For measurements of other samples (nitridated, TiO_*x*_N_*y*_–Ir and after EC-STAT), we used a protocol that can serve as a sample stability estimation. Namely, we measured single Raman spectra sequentially at a certain site using increasing laser powers of 0.6, 1.4, 3.4, 7.3, and 13.5 mW. Each sample on the TEM grid was examined at 3 sites. At higher laser powers, the samples suffered degradation, but the extent of degradation can be taken as a measure for the stability of the sample. The results are shown in the Supporting Information in Figure S2.

#### X-ray Photoelectron Spectroscopy

XPS was conducted on a PHI-TFA XPS spectrometer (Physical Electronics Inc) with an Al-monochromatic source. Surface composition calculations were performed without considering carbon, assuming its presence resulted from contamination.

#### Electrochemical measurements

A modified floating electrode (MFE) setup allowing the usage of a TEM grid as a WE was employed for electrochemical measurements. The setup’s assembly is described in detail in our recent publication (see also Figure S3).^13,22^ Briefly, the main MFE’s characteristic is the configuration of the WE electrode, which is placed on the surface of the electrolyte. Electrochemical experiments were performed in a two-compartment Teflon cell (H—cell) separated by a Nafion membrane (Nafion 117, FuelCellStore). WE and reference electrodes (reversible hydrogen reference, HydroFlex, Gaskatel) were placed separately from the Pt mesh counter electrode (CE, GoodFellow 50 × 50 mm).

Electrochemical treatment and subsequent IL-TEM analysis were conducted for TiO_*x*_N_*y*_–Ir and TiO_*x*_N_*y*_ grids. Initially, voltammetry (300 mV s^–1^, 0.05–1.45 V) was performed to resolve fingerprint iridium redox features. Afterward, OER activity was determined by linear sweep voltammetry (LSV) (20 mV s^–1^) and normalizing the current response per iridium surface charge. For this purpose, characteristic Ir(III/IV) redox peak between 0.6 and 1.1 V is integrated.^23−27^ Degradation experiments (*i.e.,* EC-STAT) were performed *via*, *i.e.*, twenty 5 min potentiostatic intervals (1.55 V) interrupted by 2 min resting steps (0.78 V). Afterward, OER activity was re-evaluated. Ohmic drop compensation (85% was compensated for) was applied during activity measurements *via* the positive feedback mode.

## Results and Discussion

### *Nano Lab* Concept

**Figure 2:**
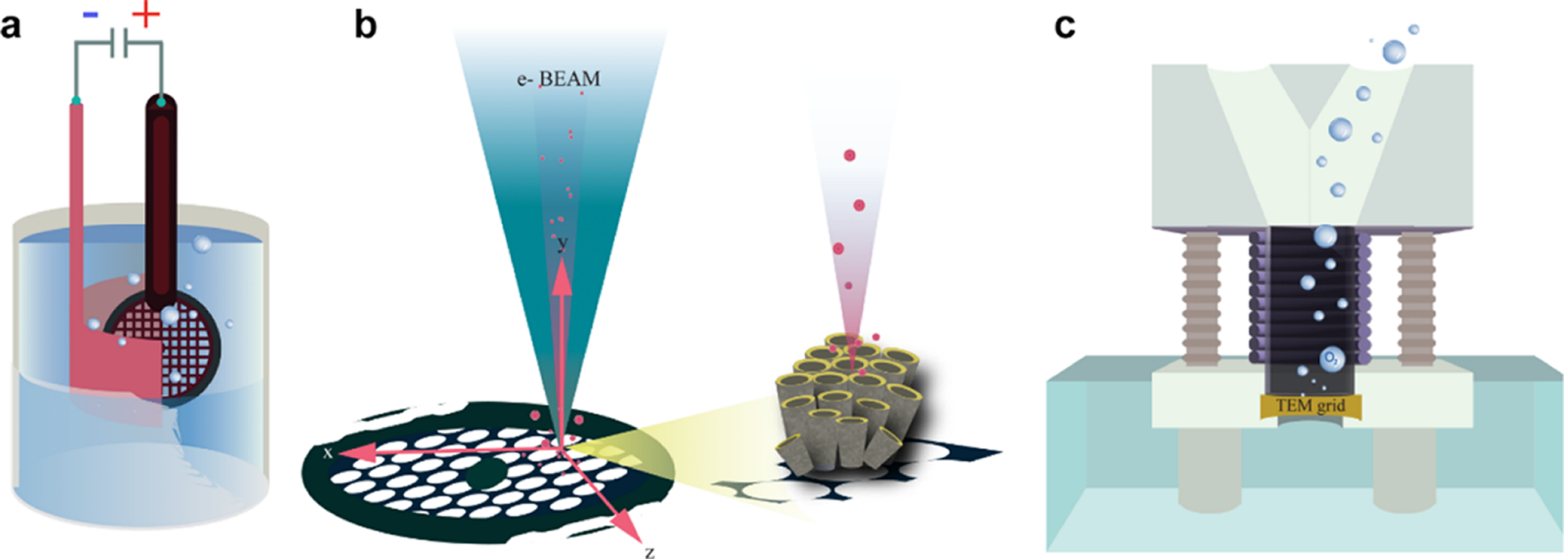
Nano Lab concept connects at least three advanced techniques: (a) anodic oxidation of the TEM grid, (b) IL-TEM technique, and (c) electrochemical characterization with the MFE setup.

Figure 2 schematically depicts the *Nano Lab* concept introduced in the present paper. The main idea is based on TEM grid exploitation as a substrate for catalyst synthesis and characterization resolved at the nanoscale level and with atomic resolution. To achieve this, the use of three equally important techniques was exploited: (i) anodic oxidation of the TEM grid as the main synthesis method, (ii) electrochemical characterization of the TEM grid-based floating electrode, and (iii) the IL-TEM technique as the main morphological and structural characterization method. As demonstrated, other methods complementary to the three main techniques include exploitation of IL-SEM, XPS, and Raman. We note that other advanced characterization methods could potentially be used as well if special cells that allow TEM grid mounting are designed.

Implementation of IL-TEM characterization demands fulfilling several prerequisites. First, the catalyst layer needs to be strongly attached to the TEM grid substrate so that it can be studied in each synthesis and characterization step. Second, the electric conductivity and mechanical stability of the grid need to remain sufficient throughout prolonged electrochemical testing. Third, the electrocatalytic film has to be thin enough in order to be transparent to the electron beam. When all these requirements are met, local structural and compositional changes of the catalyst at an identical location *before* and *after* consecutive treatments *via* TEM analysis are possible.

**Figure 3:**
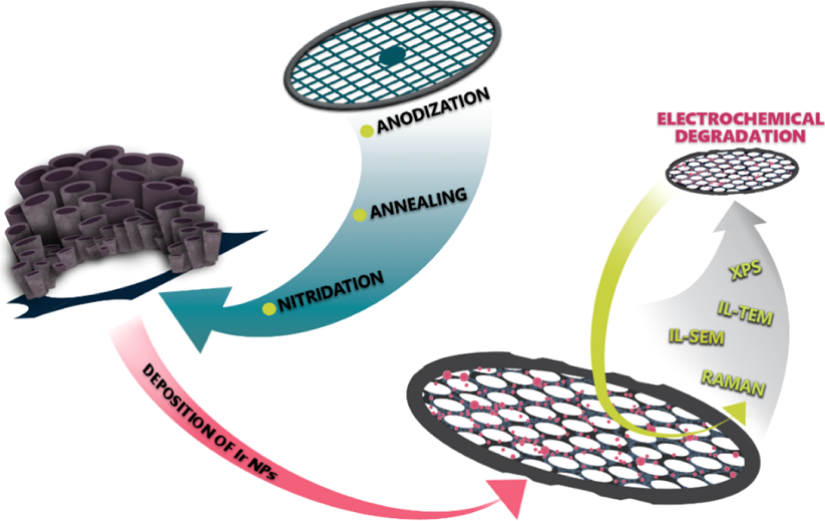
Synthesis and characterization of the TiO_*x*_N_*y*_–Ir floating electrode catalyst.

For the purpose of this study, we anodized Ti TEM grids, which served as strongly attached high-surface-area support on which the synthesis of a TiO_*x*_N_*y*_–Ir OER catalyst and subsequent electrochemical characterization were conducted. We performed identical location TEM and SEM analyses throughout the entire synthesis and characterization process and applied XPS, Raman, and electrochemical analysis to thoroughly describe the catalyst and its preparation. Figure 3 illustrates all the synthesis and characterization steps conducted to develop the TiO_*x*_N_*y*_–Ir electrocatalyst with the *Nano Lab* approach.

Anodization of the Ti TEM grid was performed with a dedicated anodic oxidation apparatus^20^ that enabled the preparation of a high-surface-area TiO_2_ immobilized film directly on the TEM grid. Anodic oxidation is the method of choice due to many important advantages, including the possibility to grow strongly attached nanostructured films directly on the TEM grid and the simplicity of the process. Additionally, the anodization is beneficial since it minimizes the chance of grid breakage during the experiments. However, the anodization conditions have to be optimized to grow a high-quality film. The most important parameters are the electrolyte composition, anodization voltage, and anodization time. Different grid mesh shapes and sizes can be anodized, influencing the anodized support surface area and the largest possible thickness of the anodized film. The largest thickness is the same as the TEM grid wire diameter; however, in this case, the film’s mechanical properties are greatly deteriorated. In the present paper, the 400-mesh titanium grid was anodized at 40 V for 30 min in an anodization electrolyte consisting of 0.3 wt % NH_4_F and 2 vol % deionized water in ethylene glycol (see Experimental Section for details), which resulted in an immobilized nanotubular, amorphous TiO_2_ film. The latter transforms into an anatase phase *via* subsequent annealing at 450 °C in air. Afterward, annealing treatment under an ammonia atmosphere is performed to convert the anatase layer to an electrically conductive TiO_*x*_N_*y*_ phase. However, this conversion has to be precisely controlled to increase electric conductivity but at the same time preventing excessive nitridation. The latter causes low mechanic stability of the grid; hence, its transfer between experimental steps (*i.e.*, the core characteristic of the *Nano Lab* concept), is difficult. Here, the conversion of amorphous TiO_2_ to the anatase phase prior to the nitridation step is instrumental for achieving a mechanically stable TiO_*x*_N_*y*_ on a Ti TEM grid composite.

The next constituent part of the *Nano Lab* is the unique electrochemical analysis enabled by the floating-type electrode setup, where the TEM grid serves as the WE (*i.e.*, modified floating electrode setup—MFE).^28^ Its main characteristic is that the active catalyst is placed on the surface of the electrolyte solution. Besides obtaining a well-resolved electrochemical signal, using this setup for the investigation of gas-evolving reactions (*i.e.*, OER) is beneficial as it also enables more efficient bubble management. This particular capability is instrumental for meaningful OER studies since bubble management has been recognized as a decisive factor for adequate prediction of catalyst lifetime. Here, an ongoing OER eventually induces oxygen bubbles and blockage of the catalyst’s surface. Even though oxygen bubbles are to some extent removed by diffusion within the catalyst layer or by convection in the radial direction at the outer surface of the catalyst layer (in the case of rotating electrodes), the remaining bubbles significantly protect the catalyst from degradation.^29,30^ This leads to erroneous conclusions on catalyst lifetime prediction, *i.e.*, an underestimated deterioration rate.^15,31^ Furthermore, evolving bubbles can cause changes in ohmic resistance during the measurement, leading to incorrect *IR*-drop compensation. Additionally, bubbles can also impede post-mortem structural characterization. Note, if the bubble-induced surface blockage is present, possible degradation mechanisms induced by the electrochemical operation can be inhibited, and hence potentially relevant insights can be overlooked. To overcome this in MFE measurements, argon can be purged on the top part of the WE, which rapidly shifts chemical equilibria towards oxygen bubble dissolution in a thin layer of electrolyte. As shown herein, coupled with a specially selected electrochemical protocol, this enables the effective removal of oxygen bubbles accumulated in a thin liquid layer.

### Characterization of the Floating Electrode

#### Morphology, Composition, and Structure of the Floating Electrode

**Figure 4:**
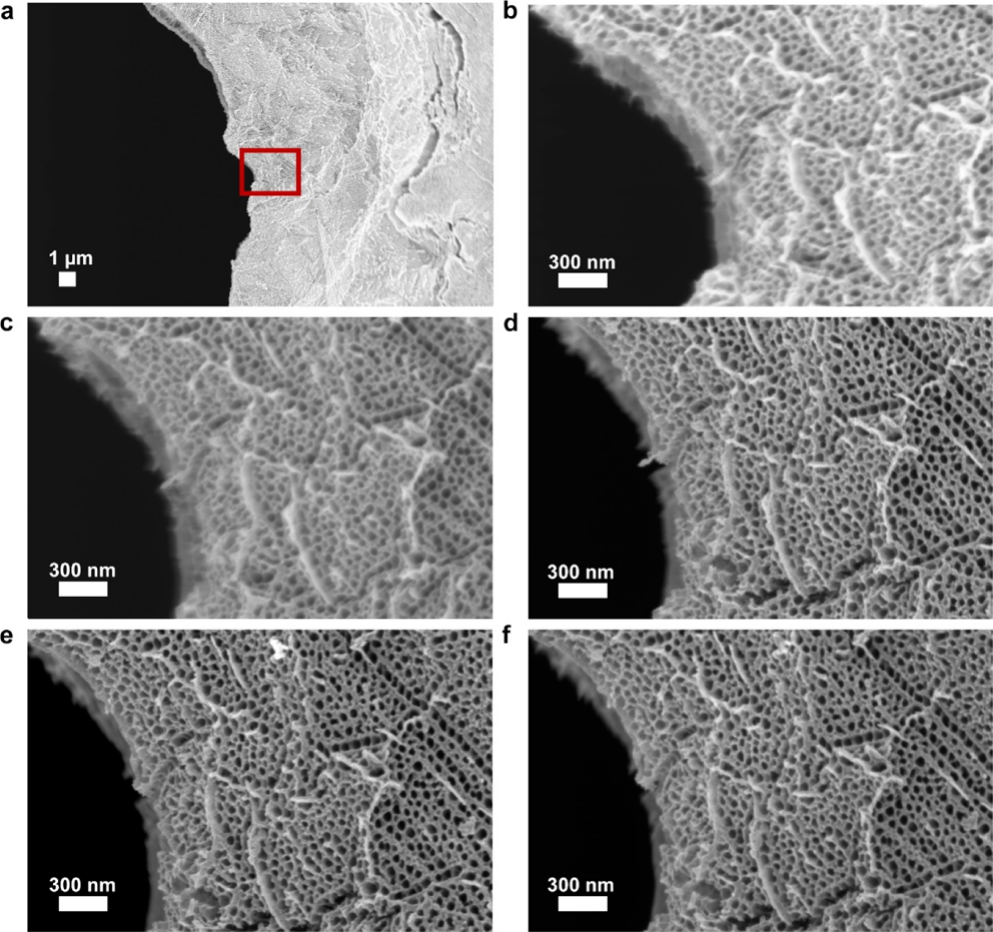
Low magnification SEM images of identical locations. (a) images at every stage of the experiment: (b) anodization, (c) annealing in air, (d) nitridation in ammonia, (e) addition of Ir nanoparticles through reduction of Ir salts, and (f) electrochemical test (EC-STAT protocol).

SEM images of the TEM grid-based sample were taken at identical locations to analyze the morphology of the TiO_*x*_N_*y*_ support in every synthesis step (Figure 4b–f). Evidently, nanotubular structure evolves during anodic oxidation; however, changes in the sample’s morphology are barely noticeable with IL-SEM analysis. The optical microscope photographs (Figure S5) of each synthesis step of a TEM grid sample demonstrate changes in the grid’s appearance. Low-magnification SEM images of the TEM grid before and after anodization (Figure S6) show that the formation of TiO_2_ slightly decreases the TEM grid hole size.

**Figure 5:**
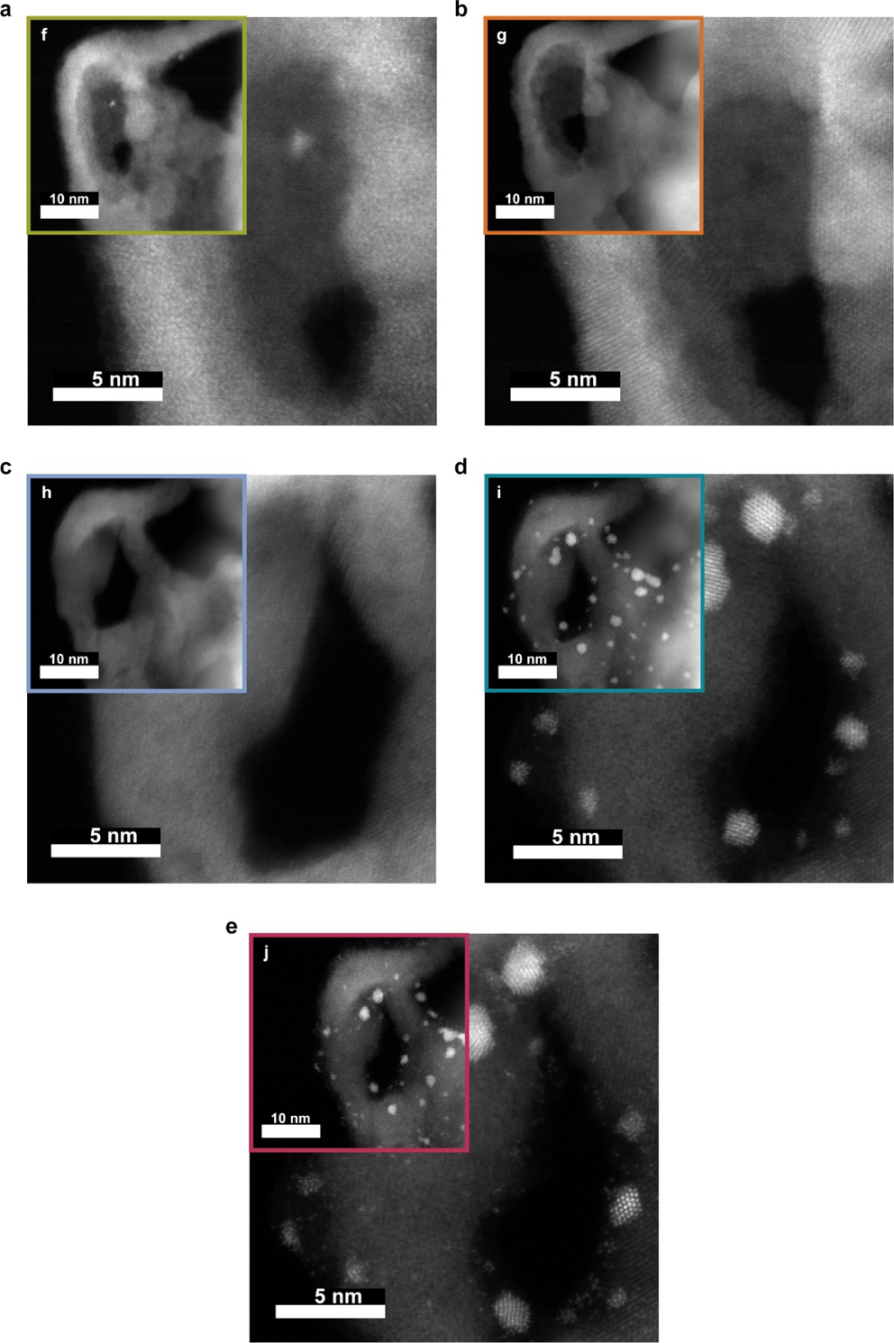
Identical location STEM–HAADF images at every stage of the experiment: (a) anodized sample, (b) air-annealed sample, (c) nitridated sample, (d) sample with deposited Ir nanoparticles, and (e) electrochemical test (EC-STAT protocol). Insets (f—j) in each image show the same spot at a lower magnification.

Further investigation was performed with IL-TEM analysis. This reveals that even the slightest changes imposed by the individual experimental step can be precisely monitored (Figure 5a–j). This is most evident in the case of the air annealing step, where TiO_2_ undergoes a structural change from amorphous to anatase phase^32^ (Figure 5, the change from a to b). In the subsequent nitridation step (formation of TiO_*x*_N_*y*_), evident restructuring occurs and surface area increases, as indicated in Figure 5b,c due to a change in the crystal structure imposed by the replacement of O with N and partial reduction of a portion of Ti ions from 4+ to 3+. The last synthesis step, Ir deposition, results in the formation of NPs (1.53 nm^2^ ± 1.43 nm^2^) and the reduction of TiO_*x*_N_*y*_ edge sharpness, as can be seen in STEM images (Figure 5c,d).

A detailed analysis of the TEM grid’s surface composition after each experimental step was performed as well. Herein, we focused on the nitrogen and oxygen distributions determined by XPS, IL-EDXS, and EELS. The elemental composition of the grid is shown in Table S1. Note that the observed differences in the N/O ratio (Table 1) are due to the different analysis depths of the characterization techniques. XPS analysis depth is 3–5 nm, and the analysis spot is approx. 0.4 mm in diameter. EELS analysis depth is 5–30 nm, whereas EDXS analysis depth is approx. 20 nm. Based on the results, the anodized TEM grid prior and after air annealing does not match the TiO_2_ stoichiometry (Table S1) and is closer to the TiO_3_ stoichiometry. We note that the excess of O may be attributed to surface contamination. After nitridation, the surface composition of 51 at % of O, 23 at % of N, and 26 at % of Ti (approx. Ti1–N1–O2) was determined. Considering the N/O ratio of 0.53, approx. one-half of the oxygen atoms in the TiO_2_ structure were replaced by nitrogen. Furthermore, in the XPS spectra (Figure S7), a characteristic N 1s peak typical for nitride and/or oxynitride phases appears at 396 eV, accompanied by two new Ti peaks at 456.0 eV (19%) and 457.2 eV (31%), characteristic for Ti-(O,N) and Ti–N, respectively. This indicates a non-uniform phase composition of the support. In the next step, *i.e.*, during the formation of Ir nanoparticles (thermal reduction of IrBr_3_), the N/O ratio slightly decreases (Table 1). This is circumstantial evidence of TiO_*x*_N_*y*_ support being the reservoir for the evolution of some nitrogen species during the reduction of Ir salts. According to XPS spectra analysis, well-resolved iridium peaks are deconvoluted with the Ir(0) metallic state (60%) and Ir oxide in the Ir(4+) state (40%) (see Section SI 5).

**Table 1:** N/O Ratio at Every Sample Stage Determined with XPS, EELS, and EDXS

	N/O			
SAMPLE STAGE	XPS	EELS	EDXS location 1	EDXS location 2
amorphous TiO_2_	0.03			
anatase TiO_2_	0.02			
nitridation	0.53	0.67 ± 0.06	10.7	13.4
Ir deposition	0.42	0.53 ± 0.07	3.5	3.5
EC-STAT	0.17	0.46 ± 0.09	2.5	2.2

Complementary information obtained from IL-EELS mapping reveals that N-rich regions and O-rich regions (Figure S8a) are randomly distributed before deposition of Ir nanoparticles. Afterward (Figure S8b), the O-rich regions move to the edge of the support and its boundaries, whereas the N-rich regions emerge in thicker regions (thickness can be observed in Figure S8 row g—thicker regions are brighter). N/O ratios obtained from IL-EDXS show the same trend as EELS and XPS data, even though EDXS is less reliable for quantitative measurements of light elements (Table 1).

#### Electrochemical Characterization *via* MFE

**Figure 6:**
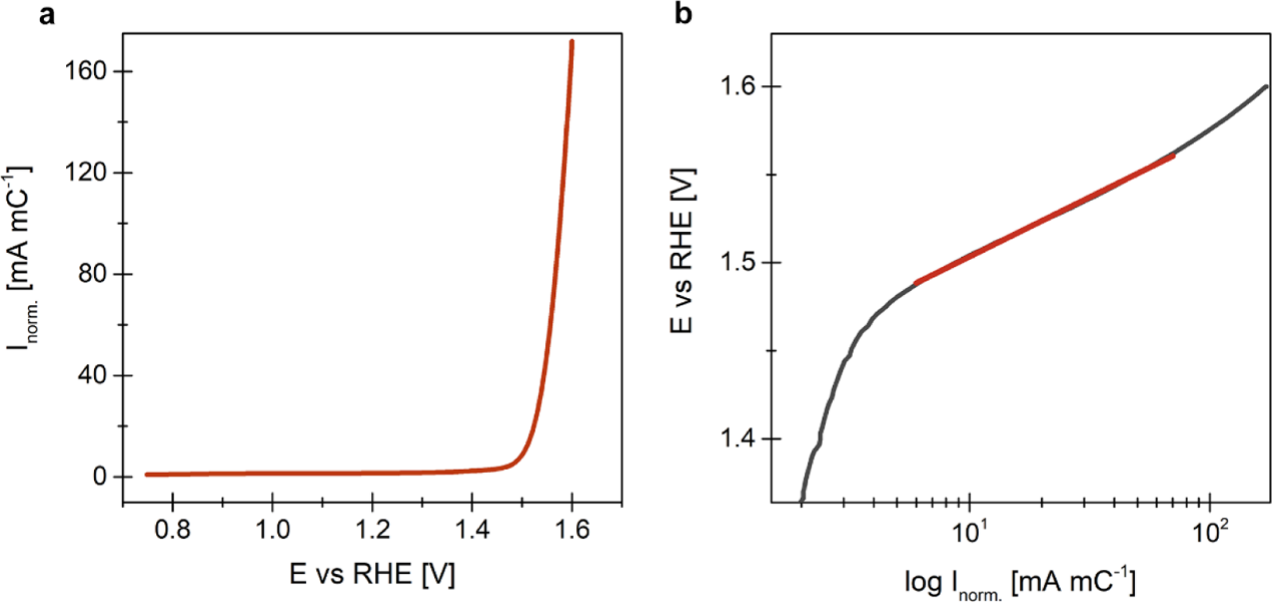
(a) Surface Ir charge normalized OER polarization curve. (b) Tafel plot of the OER polarization curve (constructed from a).

Initial electrochemical experiments focused on verifying whether satisfactory electric contact is achieved under the MFE configuration. The expected characteristic electrochemical features of iridium and OER response were targeted. Initially, a well-resolved characteristic Ir feature was obtained under fast cyclovoltammetric conditions (Figure S3c). Afterward, the OER performance was determined with LSV measurement (20 mV s^–1^) to place this particular TiO_*x*_N_*y*_–Ir in the context of OER state-of-the-art. Note that the OER polarization curve was normalized per iridium surface charge (obtained from prior fast cyclovoltammetry, see Experimental Section), as this charge is considered to have a direct relation to the number of active sites.^23−27^ Additionally, this measure eliminates the contribution of the capacitive current originating from the TiO_*x*_N_*y*_ support.^33^ The obtained value of surface-charge normalized specific OER activity (extrapolated at 1.55 V *vs* RHE, Figure 6a) is in good agreement with the previous report on TiO_*x*_N_*y*_-based analogues with similar Ir particle size.^18^ An expected Tafel slope of approximately 67 mV dec^–1^ was determined (Figure 6b). Note that this value is comparable to the literature values for typical Ir-based catalysts^34−37^ as well as TiO_*x*_N_*y*_-supported Ir analogues.^18,38,39^ Overall, the obtained OER characteristics give confidence that proper electric contact is enabled in the MFE setup.

To ensure feasible long-term electrochemical testing, we first investigated the extent of accumulated O_2_ bubbles on electrochemical response. For this purpose, a preliminary TiO_*x*_N_*y*_–Ir TEM grid sample was subjected to a potentiostatic perturbation of 30 min at 1.6 V *vs* RHE. Vacuum suction was applied to the non-electrolyte side of the WE with the purpose of securing sufficient bubble removal during OER, as demonstrated recently (Figure S4b).^15^ However, this particular mode of operation (vacuum suction) did not enable sufficient removal of O_2_ bubbles, as evident from the corresponding electrochemical response. This is gradually decreasing and eventually approaches the current response of the bare TiO_*x*_N_*y*_ analogue (Figure S4a). Nevertheless, as demonstrated herein, the detrimental effect of O_2_ bubbles can be controlled by employing a transient electrochemical bias between OER and OCP conditions (see detailed discussion in Section SI2).

#### Electrochemistry and IL-TEM Coupling

**Figure 7:**
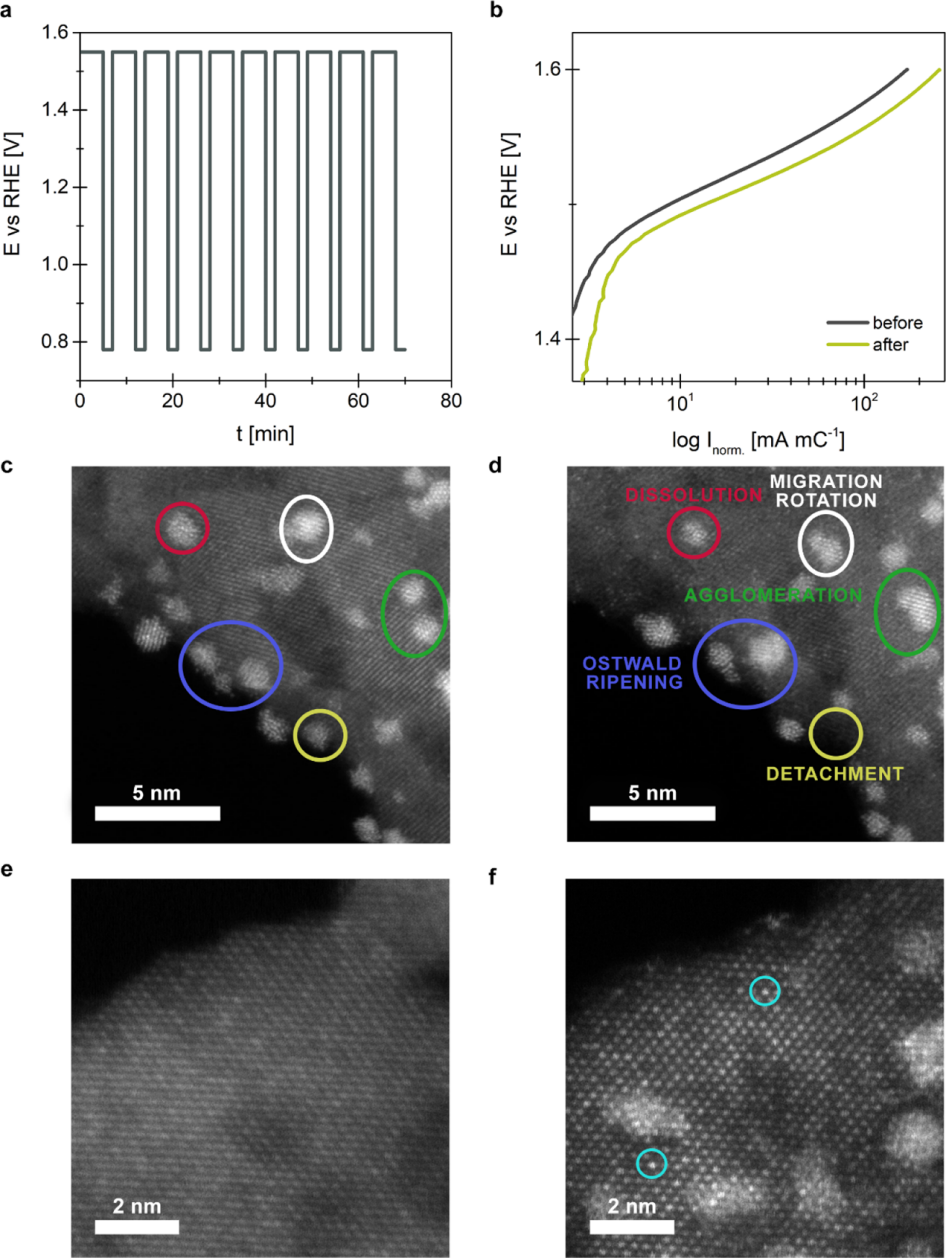
(a) Potential-time diagram used for EC-STAT electrochemical biasing. The initial 10 sequences (out of 20 altogether) are shown. (b) Tafel plots of OER polarization curves before and after EC-STAT. (c) Identical location STEM–HAADF image of the TiO_*x*_N_*y*_–Ir sample before electrochemical degradation and after it (d) showing all the possible degradation processes in a single image pair. (e) Identical location of the STEM–HAADF image of the TiO_*x*_N_*y*_–Ir sample before the addition of Ir and (f) after EC-STAT showing Ir nanoparticles as well as Ir single atoms.

Further work focused on inspecting the applicability of the sequential coupling of electrochemistry and IL-TEM analysis. We note that the goal here was not to perform a long-term performance evaluation. Rather, our target was to use a short electrochemical protocol to induce potential nanoscale events, in a relatively timely manner, *i.e.*, trigger structural changes of the TiO_*x*_N_*y*_ support and Ir nanoparticles. Ideally, this could then be well resolved *via* TEM techniques, demonstrating the applicability of the *Nano Lab* platform. Capitalizing on initial electrochemical testing (see Section SI2), a pulsed protocol referred to as EC-STAT was selected and consisted of potentiostatic intervals at 1.55 V, interrupted with 2 min steps at non-OER potentials (0.78 V, see Section Electrochemical measurements in the Experimental Section). Compared to the protocol described in the SI, we continued here with the pulse-based protocol, which proved to be feasible for the prolonged OER experiments, but chose here potentiostatic rather than galvanostatic pulses. This allowed us to gain control over the potential during the electrochemical perturbation. The upper potential limit is close to the potential at 5 mA cm^–2^, and lower potential limit was the potential at the OCP, determined at the beginning of the electrochemical experiment. Insights into nanoscale dynamics were pursued by a detailed structural characterization of pre-selected locations before and after the EC-STAT. Initially, modifications of Ir NPs were followed, where closer inspection under identical location mode highlights several examples of the ongoing degradation mechanism during electrochemical operation (Figure 7c,d). Among these, agglomeration, detachment, Ostwald ripening, and the formation of Ir single atoms (visible in Figure 7f) seem to be the prevailing mechanisms induced by EC-STAT. Interestingly, these mechanisms did not significantly alter OER performance in the time window of the experiment (Figure 6). The activity trend is also in good agreement with statistical analysis from identical location STEM images. More specifically, less than 1% of Ir particles are lost during EC-STAT, whereas other surface area indicators, *i.e.*, particle size distribution, average particle size, average nearest neighbor distance, and average circularity, stay roughly unaltered (Table S2, Figure S10). On the other hand, Ir composition clearly changes as evident from XPS analysis, where after EC-STAT biasing, the Ir 4f spectrum still shows the presence of two oxidation states of Ir, but the Ir(4+) state is 70% and the Ir(0) presents 30% of total Ir atoms (see Section SI 5, Figure S6c). Note that this is in line with the formation of surface/subsurface Ir(4+) oxide in this particular potential window.^40−43^ Accordingly, the slightly increased activity could be ascribed to electrochemically formed, more active, amorphous Ir oxide.^44^ Note that its evolution is favored during electrochemical perturbation in a wide potential window.^45^ The composition of TiO_*x*_N_*y*_ support changes as well during electrochemical perturbation according to the EELS, XPS, and IL-EDXS analyses, revealing a decrease in the N/O ratio (Table 1). We would like to highlight that the EELS values are within the measurement’s uncertainty, hence, ascribing the obtained trend to electrochemical oxidation of TiO_*x*_N_*y*_ is at this point unreliable. To get more solid evidence, the results were compared with a separate, bare TiO_*x*_N_*y*_ grid sample. The comparison indicates a significant change in the N/O ratio after electrochemical perturbation for the bare TiO_*x*_N_*y*_ sample from 0.75 ± 0.13 to 0.23 ± 0.06, which directly confirms that TiO_*x*_N_*y*_ gets electrochemically oxidized. This is also in compliance with the redox behavior observed in a separate investigation, which reveals irreversible oxidation of TiO_*x*_N_*y*_ (Figure S12a). However, more intriguing is the significantly smaller N/O ratio decrease for the TiO_*x*_N_*y*_–Ir analogue, which implies that Ir might have a protective effect on electrochemical oxidation of the TiO_*x*_N_*y*_ support. The decrease in the N/O ratio is in line with the XPS analysis, where the relative amount of TiO_2_ increased from 50 to 65% at the expense of the Ti(O, N) and Ti–N bonds. A similar trend in N/O ratios was also obtained *via* EDXS characterization of identical locations (Table 1). The stabilizing effect of Ir could provide a relevant means to stabilize supported OER catalysts; however, further efforts are required to elucidate this interaction. Here, especially in regards to the electric conductivity of the TiO_*x*_N_*y*_ support, the N/O ratio should be considered a relevant parameter in future efforts. The altered electric conductivity, would, however likely play a role during long-term operation since it does not seem to play a role within the timeframe of EC-STAT, where the OER activity even increases (see Figure 7b).

## Conclusions

To conclude, the *Nano Lab* concept represents an innovative approach to the nanoscale understanding and development of nanostructured materials. We show its use, capabilities, and advantages using the example of a novel synthesis and advanced analysis of an OER nanoparticulate electrocatalyst. Ti TEM grid was anodically oxidized for the first time with an in-house developed anodization apparatus, calcined in air, and nitridated in ammonia to form a TiO_*x*_N_*y*_ nanotubular film on a Ti TEM grid, which provided a support for the Ir electrocatalyst. The TiO_*x*_N_*y*_–Ir electrocatalytic composite was electrochemically treated and analyzed using a floating electrode setup. The process was followed with IL-TEM and other *ex situ* techniques (IL-SEM, XPS, and Raman) at every step of the synthesis and electrochemical treatment. The new *Nano lab* concept offers a general platform for the development of a wide range of catalytic materials and applications and enables new insights into the relation between structure and composition on the atomic level and catalytic analysis.

## Supporting Information Available

Raman spectroscopy results; details on the MFE setup and bubble management; analysis of TEM grids with an optical microscope; elemental composition of all samples analyzed with XPS, EELS, and EDXS; detailed XPS results; IL-EELS mapping images; additional SEM and TEM analyses of the floating electrode; detailed analysis of Ir nanoparticles; and additional electrochemical results (PDF)
