# Supplementary material for: “Nano Lab” Advanced Characterization Platform for Studying Electrocatalytic Iridium Nanoparticles Dispersed on TiOxNy Supports Prepared on Ti Transmission Electron Microscopy Grids
Source: ACS Appl Nano Mater. 2023 Jun 5;6(12):10421–30. doi: 10.1021/acsanm.3c01368 (PMC10294127; doi:10.1021/acsanm.3c01368)
Supplement: Supplementary file 1 — an3c01368_si_001.pdf [file an3c01368_si_001.pdf]

SUPPORTING INFORMATION

"*Nano Lab*" Advanced Characterization Platform for Studying Electrocatalytic Iridium Nanoparticles Dispersed on TiO_xN_y Supports Prepared on Ti Transmission Electron Microscopy Grids

Marjan Bele,[1, ∇,*], Gorazd Koderman Podboršek,[1,2, ∇], Anja Lončar, [1,3] Primož Jovanovič,[1,*] Armin Hrnjić,[1,3] Živa Marinko,[2,4] Janez Kovač,[5] Angelja Kjara Surca,[1] Ana Rebeka Kamšek,[1,6] Goran Dražić,[1,2] Nejc Hodnik[1,2], Luka Suhadolnik[4,7,*]

[1] Department of Materials Chemistry, National Institute of Chemistry, Hajdrihova 19, SI-1000 Ljubljana, Slovenia

[2] Jožef Stefan International Postgraduate School, Jamova 39, SI-1000 Ljubljana, Slovenia

[3] University of Nova Gorica, Vipavska 13, SI-5000 Nova Gorica, Slovenia

[4] Department for Nanostructured Materials, Jožef Stefan Institute, Jamova 39, SI-1000 Ljubljana, Slovenia

[5] Department of Surface Engineering and Optoelectronics, Jožef Stefan Institute, Jamova 39, SI-1000 Ljubljana, Slovenia

[6] Faculty of Chemistry and Chemical Technology, University of Ljubljana, Večna pot 113, SI-1000 Ljubljana, Ljubljana

[7] Department of Chemical and Pharmaceutical Sciences, University of Trieste, via L. Giorgieri 1, 34127 Trieste, Italy

* Corresponding authors: Dr. Marjan Bele, e-mail: marjan.bele@ki.si

Dr. Primož Jovanovič, e-mail: primoz.jovanovic@ki.si

Dr. Luka Suhadolnik, e-mail: luka.suhadolnik@units.it

SI 1. Raman spectroscopy

Anodized/air-annealed state: The air-annealed sample on grid was extremely sensitive to laser light which may be due to the small size of nanoparticles. The changes of the bands occurred slowly during recording with increasing number of scans (**Figure S1a, b**). When using 20 scans, the characteristic spectrum of anatase prevailed: sharp band at 142 cm^{-1} with a shoulder band at 197 cm^{-1} and the triple bands at 399 cm^{-1}, 516 cm^{-1} and 633 cm^{-1}. They correspond to the bands of anatase monocrystal at 144 (E_g) cm^{-1}, 197 (E_g) cm^{-1}, 399 (B_{1g}) cm^{-1}, 513 (A_{1g}) cm^{-1}, 519 (B_{1g}) cm^{-1} and 633 (E_g) cm^{-1} as determined by Ohsaka et al.[46] (**Figure S1a**). With increasing number of scans, at some sites the spectrum of rutile is obtained (**Figure S1b**). The modes of rutile for monocrystal were reported at 143 (B_{1g}) cm^{-1}, 447 (E_g) cm^{-1}, 612 (A_{1g}) cm^{-1} and 826 (B_{2g}) cm^{-1} [47]. Since gradual transformation of modes was noted during the measurements, such results indicated that laser induced the transformation of anatase to rutile. The air-annealed sample, however, is characterised by the presence of anatase.

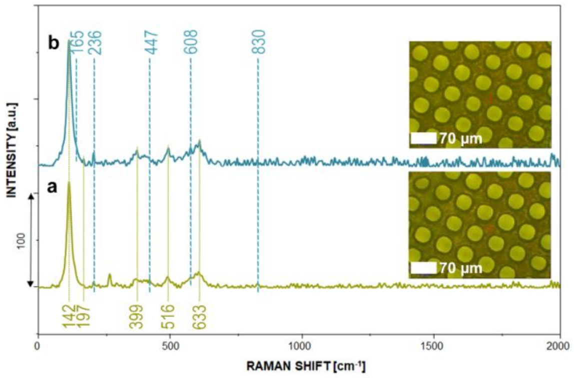

Figure S1: Raman spectra of the air-annealed sample deposited on the TEM grid on two different sites: **a)** 100 scans and **b)** 20 scans. Olive lines depict anatase bands and blue lines rutile bands. Red crosses in the optical images depict the measurement sites.

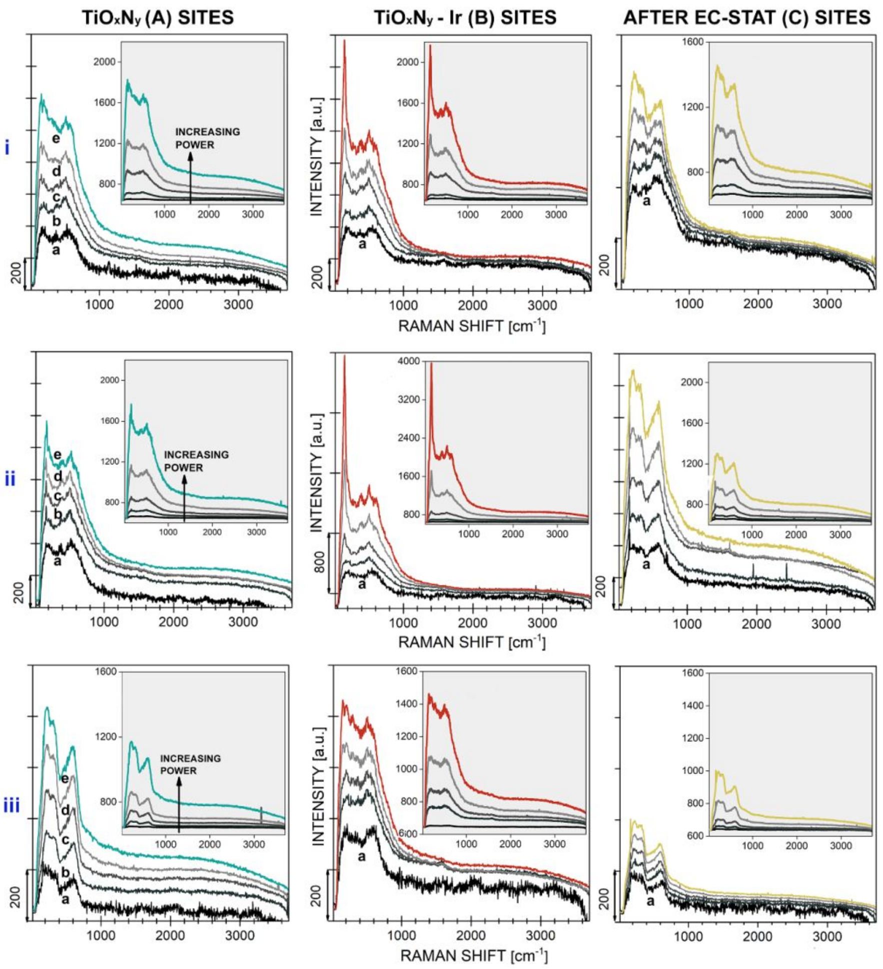

Figure S2: Raman spectra were recorded on three sites on TEM grid for: **A)** nitridated, **B)** TiO_xN_y–Ir and **C)** after EC-STAT states. The spectra were measured sequentially at each site using an increasing laser power of **a)** 0.6 mW, **b)** 1.4 mW, **c)** 3.4 mW, **d)** 7.3 mW and **e)** 13.5 mW. Originally measured spectra are shown in insets while, for easier comparison, the main graphs depict the enlarged spectra (various enhancements).

The Raman spectra of nitridated sample, TiO_xN_y–Ir, and after EC-STAT are shown in **Figure S2**. For each sample, the spectra were measured on three different sites (i-iii), by sequentially increasing laser power on that site. The spectra are shown in *as–measured* ratio in insets revealing their intensity increase with increasing laser power. It is obvious that some band changes occur in the *as–measured* spectra which can definitely be ascribed to the laser-induced changes of the samples. Such measurement protocol can enable conclusions about the stability of the samples (i) if made in the same way for different samples and (ii) if changes in the bands started to occur at different laser powers. The comparison of the spectra becomes easier when they are enlarged to a similar intensity (depicted in the

main graphs). The changes started gradually when laser power of 3.4 mW was employed but they differ for each sample. Understandable – since measurements with increasing laser powers are only an approach towards the estimation of the stability of the samples – only Raman spectra recorded with the low laser powers can be used for the interpretation of the sample structure.

The spectra (i-iii) of the nitridated sample recorded with 0.6 mW reveal a broad group of bands between 100 cm^{-1} and 800 cm^{-1} (**Figure S2 column (A), lines a**). The structure of these bands resembles the shape of the bands characteristic for TiN: a double band at approximately 215 cm^{-1} and 315 cm^{-1} and, a broader one between 540 cm^{-1} – 560 cm^{-1} [48]. The positions of these bands were reported to shift in dependence of stoichiometry [49]. Our spectra of the nitridated samples (0.6 mW) show the bands at 203 cm^{-1}, 288 (sh) cm^{-1}, 387 cm^{-1} and 516 cm^{-1} (i and ii states) which are slightly shifted with regard to TiN. We assume that the shifts are the consequence of the in-part presence of the oxynitride structure as shown by XPS. Unfortunately, up to date, we could not find the relevant spectra of oxynitrides in the literature. Therefore, the systematic examination of the powdered samples prepared in various oxynitride ratios is currently under investigation. The bands in the third investigated nitridated site (iii), however, are much more similar to the above-described bands of the TiN spectrum. Namely, a doublet bands were noted at 220 cm^{-1} and 312 cm^{-1} while the broad band appeared at 606 cm^{-1} with a shoulder at 548 cm^{-1}. This suggests that the site iii is the N rich region but the sites i and ii were more O rich regions on the TEM grid sample. With increasing laser power (**Figure S2 column (A)**), the bands at lower wavenumbers started to increase in intensity with regard to the bands in the region 540 cm^{-1} – 560 cm^{-1}. The shape of the bands in sites i and ii signals the tendency to the formation of anatase while the shape and positions of bands in site iii do not change. These results tentatively point that the stability of N rich regions exceeds that of O rich regions.

When TiO_xN_y–Ir was examined, the spectra recorded with 0.6 mW showed similar bands (203 cm^{-1}, 288 (sh) cm^{-1}, 382 cm^{-1} and 552 cm^{-1}) in i and ii sites (**Figure S2 column (B), lines a**). The spectrum of the third site (iii) slightly more approaches to the spectrum of TiN. When laser power was increased the spectra in i and ii sites changed in a dramatically quicker way with regard to the nitridated sample without Ir particles (**Figure S2 column (A)**). Namely, an intense band at 142 cm^{-1} developed that signalled the formation of anatase (**Figure S2 column (B)** and **rows i and ii**). The characteristic anatase triple bands (399 cm^{-1}, 516 cm^{-1} and 633 cm^{-1}) become visible as well. On the other hand, the changes on site iii are not so pronounced. The spectra (iii) recorded at higher laser powers show the beginning of transformation but the anatase bands do not develop considerably.

Electrochemical perturbation (EC-STAT): After exposure of the sample to the electrochemical treatment, the shape of the spectra recorded at 0.6 mW even more resembles the spectrum of TiN, especially on sites ii and iii (**Figure S2 column (C)**). With increasing laser power, the stability of these sites (i-iii) is approximately similar or even better than the stability of the nitridated sample (**Figure S2 column (A)**) but considerably improved compared to the TiO_xN_y–Ir sample (**Figure S2 column (B)**).

Raman measurements confirmed that two different types of sites appeared on the TEM grid sample: O rich and N rich regions. The spectra of the N rich regions possess more characteristics of the TiN spectrum (doublet low-frequency band with a broad band at ~550 cm^{-1}). The shifts of some bands in the spectra of the O rich regions are most probably induced by the characteristics of the oxynitride structure. The measurement protocol with increasing laser power enables some temptative conclusions on the stability of these sites. Namely, it is shown that N rich sites undergo smaller transformation than the O rich sites. The latter tend to transform into anatase. As this transformation is reasonably slow for nitridated sample (sites i and ii in **Figure S2 column (A)**), it becomes extremely quick for O rich sites (i,ii) in TiO_xN_y–Ir sample (**Figure S2 column (B)**). Interestingly, the spectra recorded after EC-STAT revealed N-rich sites with reasonable stability (**Figure S2 column (C)**). The described difference in stability of TEM grid states is under investigation.

SI 2. Modified floating electrode_Bubble management

In order to inspect the capability of MFE for efficient bubble management, two TiO_xN_y–Ir grids were prepared following the same procedure as described in Experimental section. Each grid was used for a designated electrochemical protocol:

- **i)** Vacuum suction was applied to the non – electrolyte side of the working electrode (WE) (**Figure S3**).

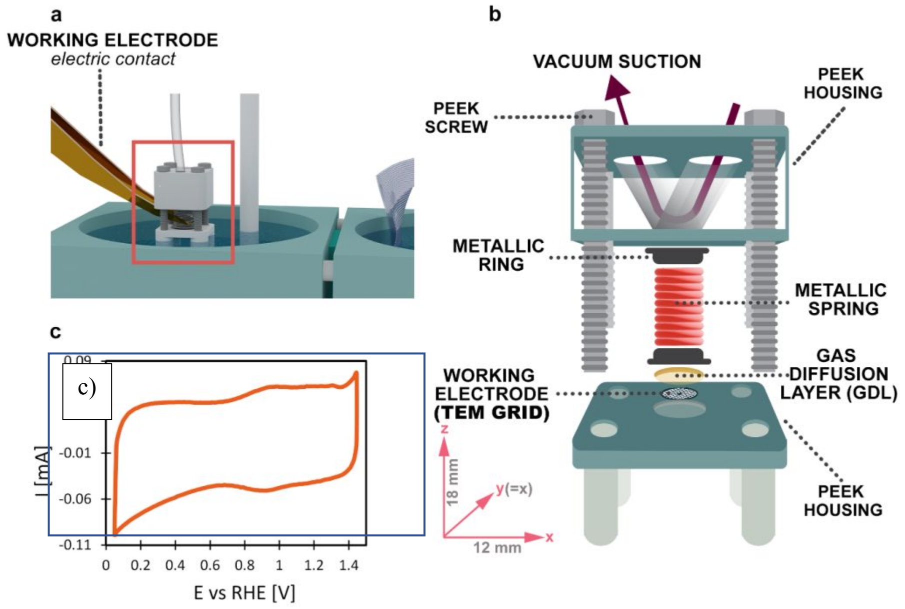

Figure S3: Schematic representation of a floating electrode setup showing **a)** the entire setup placed in H-cell and **b)** exploded view with all the parts. Vacuum suction was first applied to the non-electrolyte side of the working electrode and was later replaced with argon purging which was used for EC-STAT electrochemical protocol. **c)** 100th voltammetric cycle (300 mV s^{-1}) obtained in the electrochemical pre-treatment of TiO_xN_y–Ir TEM grid.

- **ii)** A potentiostatic – pulsed based biasing, referred from here on as EC-STAT. During the experiment, argon was purged through the system to establish non-oxygen atmosphere which turned out to be essential for efficient bubble management. EC-STAT consisted of "square-wave pulses" from 5 mA cm^{-2} to 0 mA cm^{-2}. The length of a pulse was changed during the experiment from 30 s to 300 s to test the effect of pulse duration on the accumulation of the bubbles. The exact time intervals are shown in **Figure S4b** (*x*-*y* represents *x* s at 5 mA cm^{-2} and *y* s at 0 mA cm^{-2}). The "square-wave cycling" protocol enables efficient bubble management which is evident from relatively small increase in potential during the experiment.

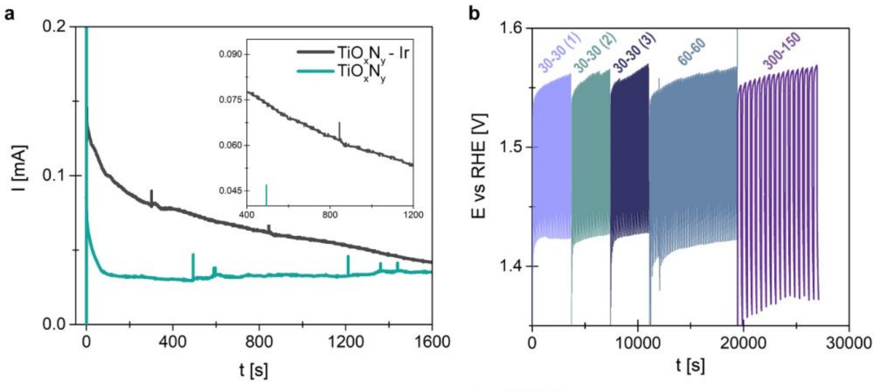

Figure S4: a) Electrochemical inspection of bubble management under vacuum suction analysed with current behaviour under chronoamperometry (1.6 V vs RHE). The magnified part of the current response demonstrates small current oscillation due to bubble removal. **b)** Variation of pulse durations; pulse consists of *x* s at 5 mA cm^{-2} and *y* s at 0 mA cm^{-2} (legend *x*-*y*).

SI 3. Optical Microscope and Scanning Electron Microscope Analysis

Images of the TEM grid taken with the optical microscope in **Figure S5** show how the sample appearance was changing with each step of the experiment. After the first synthesis step, anodic oxidation, the TEM grid appears to have a metallic silver color with a slight scent of white (**Figure S5a**). After the second step, annealing in air, the TEM grid turns into a bluish purple color (**Figure S5b**). This is typical for thin oxide films, where oxygen vacancies work as color centers. In the next step, nitridation, the color of the TEM grid changes to gold and black in most areas of the TEM grid (**Figure S5c**). This color change is attributed to the formation of titanium oxynitride, which has a gold color in thin films form and black color in nanopowder form due to high conductivity. The color does not change significantly in the subsequent two stages: i) addition of Ir nanoparticles through reduction of Ir salts and ii) electrochemical degradation experiment as can be seen in **Figure S5d** and **e**. After the third step, nitridation, the grid becomes more fragile which increases the probability of breaking. Parts of the TEM grid broken away can be seen in **Figure S5d** and **e**.

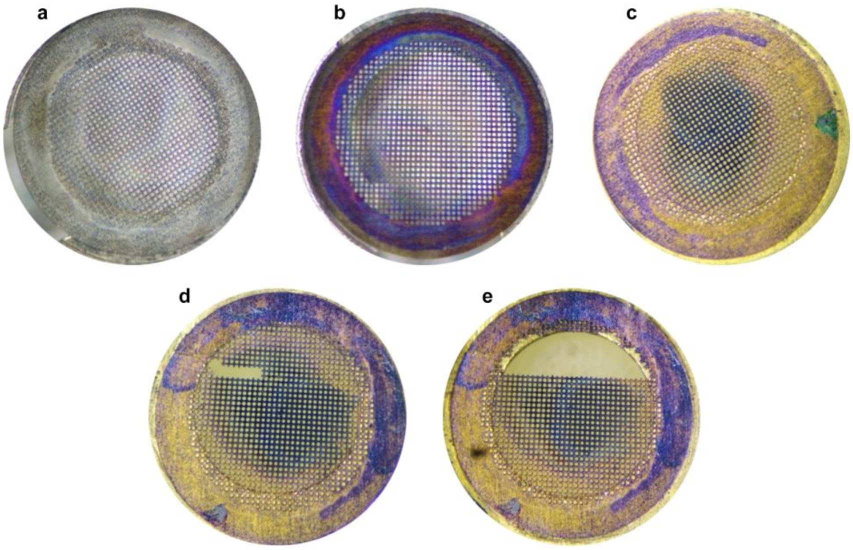

Figure S5: Optical microscope images of the TEM grid at every stage of the experiment (a – anodization, b – annealing in air, c – nitridation in ammonia, d – addition of Ir nanoparticles through reduction of Ir salts, e – electrochemical degradation experiment.

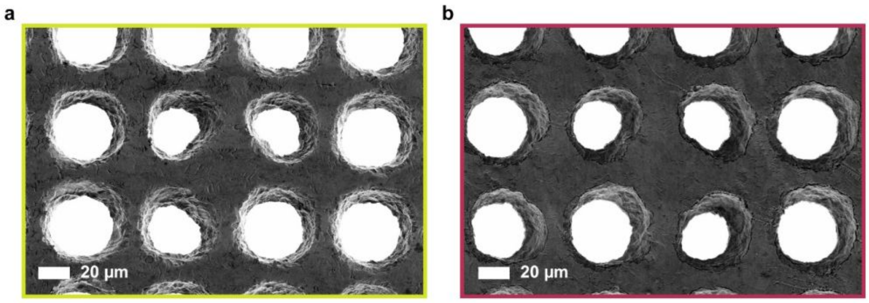

Figure S6: Scanning electron microscope images of the TEM grid **a)** before and **b)** after anodic oxidation.

SI 4. Elemental composition of samples analysed with XPS, EELS and EDXS

Table S1: Elemental composition of the sample at each experiment stage analysed with XPS, EELS and EDXS. EELS results are the average of 40 measurements at different locations. EDXS measurements were done at two locations.

	O	N (at.%)	Ti	Ir	N/O	O/Ti	N/Ti	(N+O)/Ti	$Ir^{(0)}$ of total Ir	$Ir^{(4+)}$ of total Ir
**AMORPHOUS TiO_2**										
**XPS**	*72.7*	*2*	*25.3*	*0*	*0.03*	*2.87*	*0.08*	*2.95*	*0*	*0*
**ANATASE TiO_2**										
**XPS**	*74.1*	*1.3*	*24.6*	*0*	*0.02*	*3.02*	*0.05*	*3.07*	*0*	*0*
**NITRIDATION**										
**XPS**	*51.2*	*22.7*	*26.1*	*0.0*	*0.44*	*1.96*	*0.87*	*2.83*	*0.0*	*0.0*
**EELS**	*34 ± 2*	*22 ± 1*	*44 ± 1*	-	*0.67*	*0.77*	*0.50*	*1.27*	-	-
**EDXS**	*1.47*	*15.73*	*82.28*	-	*10.70*	*0.02*	*0.19*	*0.21*	-	-
	1.18	*15.83*	*82.37*	-	*13.42*	*0.01*	*0.19*	*0.21*	-	-
**Ir DEPOSITION**										
**XPS**	*59.5*	*17.7*	*21.3*	*1.6*	*0.30*	*2.80*	*0.83*	*3.63*	*0.6*	*0.4*
**EELS**	*38 ± 2*	*20 ± 1*	*42 ± 1*	-	*0.53*	*0.90*	*0.48*	*1.38*	-	-
**EDXS**	*0.35*	*1.23*	*96.14*	*0.69*	*3.51*	*0.00*	*0.01*	*0.02*	-	-
	0.36	*1.27*	*95.61*	*0.89*	*3.53*	*0.00*	*0.01*	*0.02*	-	-
**EC-STAT**										
**XPS**	*68.8*	*13.1*	*16.6*	*1.5*	*0.19*	*4.14*	*0.79*	*4.93*	*0.3*	*0.7*
**EELS**	*40 ± 4*	*18 ± 2*	*42 ± 2*	-	*0.46*	*0.95*	*0.43*	*1.38*	-	-
**EDXS**	*0.50*	*1.24*	*96.48*	*0.76*	*2.48*	*0.01*	*0.01*	*0.02*	-	-
	0.53	*1.17*	*95.99*	*1.04*	*2.21*	*0.01*	*0.01*	*0.02*	-	-

SI 5. XPS analysis

XPS analyses were performed on Ti TEM-grid samples after annealing, oxidation, nitridation, Ir deposition, and EC-STAT. Information about the surface composition, the oxidation states, and/or the bonding of elements on the surface was obtained from the XPS data. The following peaks were detected in the XPS spectra: Ti 2p, Ti 3s, O 1s, N 1s, C 1s, K 2p, Si 2p, and Ir 4f. Surface composition considering elements O, Ti, N and Ir after different stages for TiO_xN_y–Ir was obtained and is shown in **Table S1**.

Carbon was assumed to be detected due to contamination and was therefore not taken into account. Also, elements Si and K were detected in amount of few at. %, but they were not considered for quantification. XPS results in **Table S1** show that the surface composition of anodized and air annealed sample resembles the TiO_3 stochiometry due to surface contamination. There are roughly 74 at. % of O and 25 at. % of Ti. After nitridation, N is introduced into the TiO_2 structure resulting in 51 at. % of O, 23 at.% of N and 26 at. % of Ti (approximately Ti1-N1-O2). The N/O ratio after nitridation was 0.44, meaning that approximately one half of oxygen atoms in the original TiO_2 structure was replaced by nitrogen. To get insight into surface chemistry after different sample treatments, high-energy resolution XPS spectra were acquired. **Figure S7** shows XPS spectra Ti 2p and Ir 4f after Ir deposition and after EC-STAT. Ti 2p spectra consist of the peaks Ti $2p_{3/2}$ and Ti $2p_{1/2}$ separated by 5.9 eV. On the oxidized sample, Ti $2p_{3/2}$ peak is at 458.4 eV (not shown), proving the Ti(4+) oxidation state in the TiO_2 structure. After nitridation, N 1s peak at 396 eV appears and it is related to the nitride and/or oxynitride formation and the Ti 2p spectrum exhibits a large change. In addition to Ti $2p_{3/2}$ peak at 458.6 eV of Ti(4+) (50 %), two new peaks appear at 456.0 eV (19 %) and 457.2 eV (31 %) characteristic for Ti(O, N) and Ti-N bonding, respectively [44].

After the Ir deposition, about 1.6 at. % of Ir are present on the surface. The XPS spectra of Ti 2p, O 1s and N 1s do not change significantly after Ir-deposition concerning nitridated samples. The Ir 4f spectrum consists of a peak Ir $4f_{7/2}$ at 61.4 eV and Ir $4f_{5/2}$ separated for 3.0 eV to the higher binding energy. After Ir deposition, the Ir $4f_{7/2}$ spectrum was deconvoluted in a peak at 61.4 eV related to the Ir(0) metallic state and the second peak at 62.6 eV, which is related with Ir-oxide in the Ir(4+) state [45]. From their intensities it follows that after Ir deposition about 60 % of the Ir atoms are in a metallic state and about 40 % are in Ir(4+). We should note that the Ir 4f spectrum overlaps with Ti 3s spectrum at 60 eV, which makes it difficult to quantify Ir presence. After EC-STAT Ir 4f spectrum still shows presence of two oxidation states of Ir, but the Ir(4+) state is 70 % and Ir(0) presents 30 % of total Ir atoms (**Figure S7c**). The total concentration of Ir on TiO_xN_y–Ir after EC-STAT is similar as after Ir deposition, i. e. 1.5 at. %.

Another change observed by XPS after EC-STAT is that the concentration of N decreased and O concentration increased (**Table S1**) due to the replacement of N with O during the catalytic reaction. The Ti 2p spectrum after EC-STAT (**Figure S7d**) has similar features as a spectrum after nitridation, i.e., peaks of Ti(4+) at 458.6 eV and two peaks at 456.0 eV and 457.2 eV related with Ti-O-N and Ti-N

bonds. After EC-STAT the relative amount of TiO_2 increased from 50 % to 65 % at the expense of the Ti(O, N) and Ti-N bonds, reflecting a decrease of total N concentration during EC-STAT.

N 1s spectra also underwent some changes (not shown here). In addition to the nitride peak at 396.0 eV, two peaks at 400.0 eV and 401.0 eV appeared after EC-STAT. They can be related to the transformation of the nitride bonds. They can be attributed to interstitial positions of N-atoms (peak at 400.0 eV) and the formation of N-O type species (peak at 401.0 eV) on the surface.

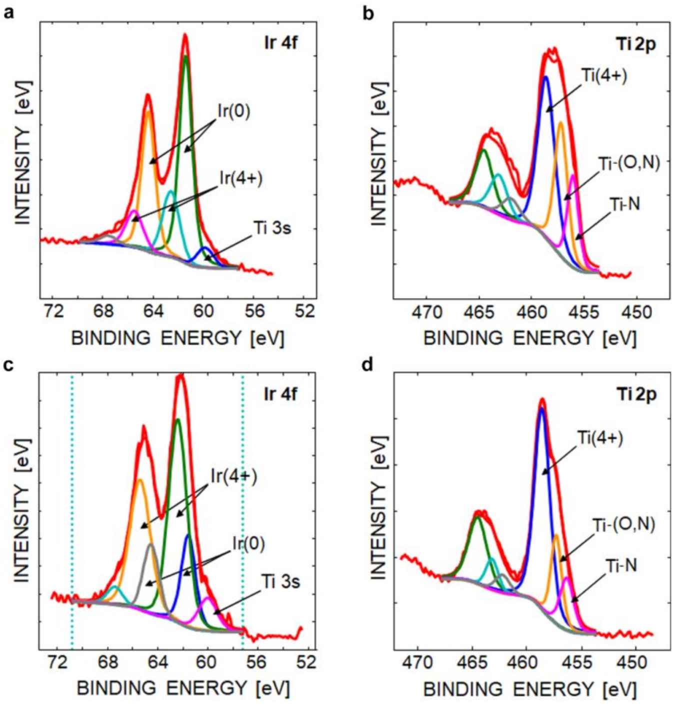

Figure S7: XPS spectra of Ir 4f and Ti 2p after Ir deposition (**a, b**) and after EC-STAT (**c, d**) for TiO_xN_y–Ir sample.

SI 6. IL-EELS mapping

Figure S8 shows results of identical location EELS mapping.

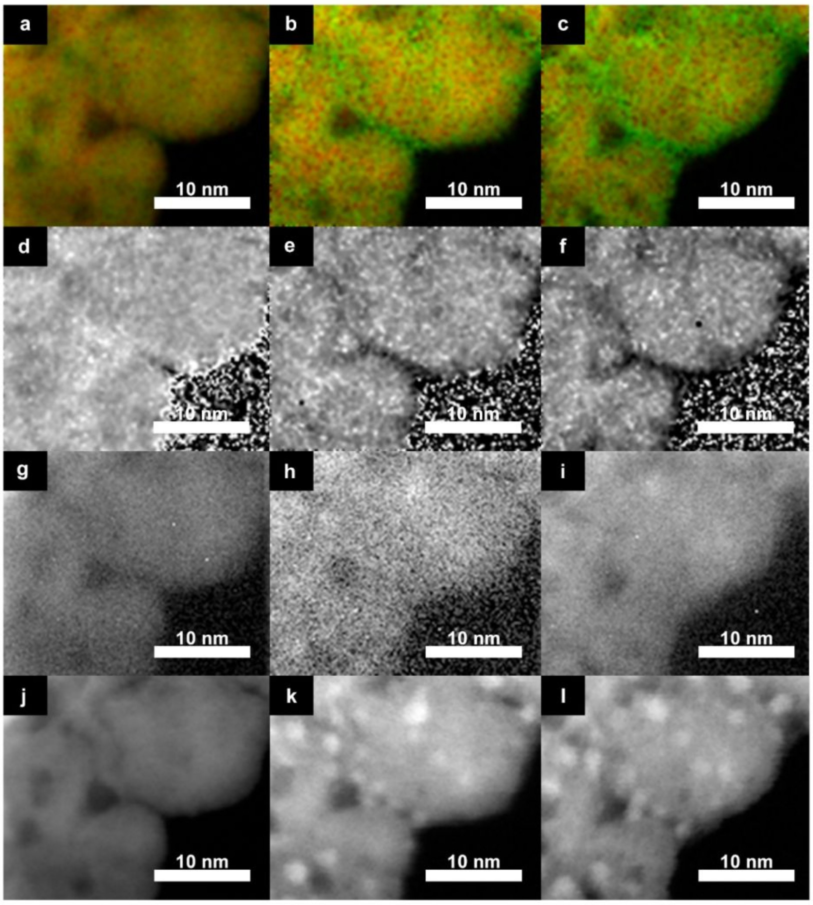

Figure S8: Identical location EELS mapping images of TiO_xN_y–Ir sample before added Ir **(column a**), before degradation (**column b**) and after EC-STAT (**column c**). **Row a** shows colour images, where red is nitrogen and green is oxygen. Images in **row d** show N/O ratio in every pixel (brighter pixel means higher N/O ratio). **Row g** shows thickness calculated from EELS. The brighter the pixel the thicker the sample in that pixel. **Row j** shows EELS high loss maps (the brightness of a pixel corresponds to the integral of the whole captured spectrum in that location).

SI 7. Additional SEM and TEM analysis of floating electrode and detailed analysis of Ir nanoparticles

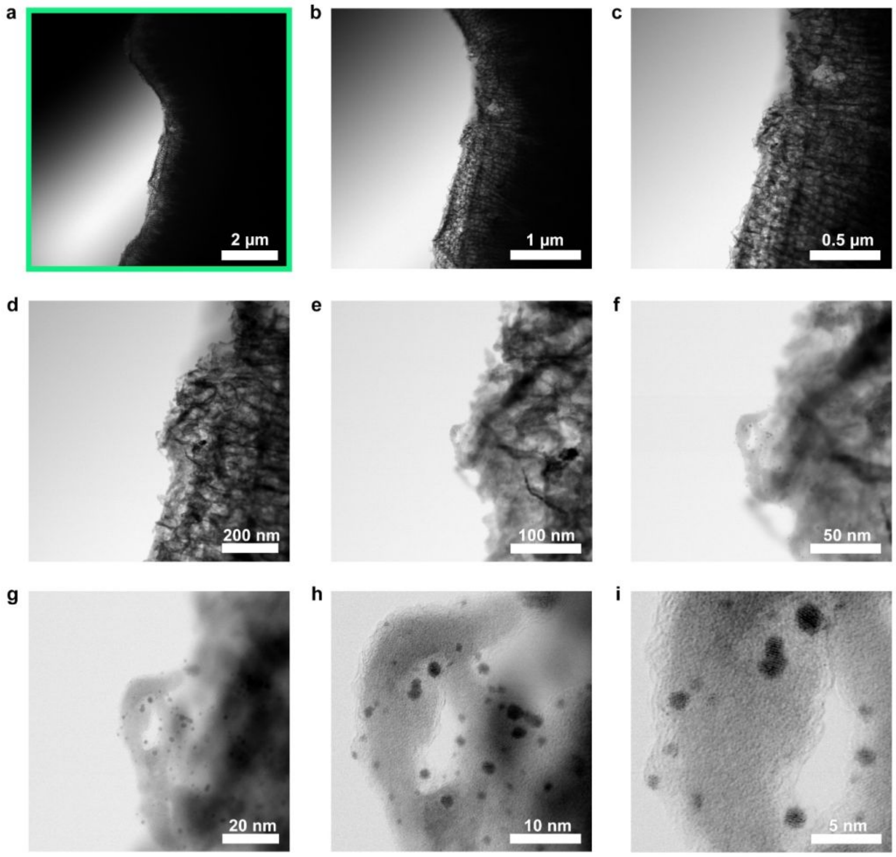

Figure S9: Scanning transmission electron microscope images of TiO_xN_y–Ir floating electrode at different magnifications. Images **b, c, d, e, f, g, h, i** are magnified 2.5x, 5x, 10x, 25x, 50x, 100x, 250x, 500x relative to image **a**, respectively. The green square in image **a** indicates the same location in SEM and TEM images (**Figure S11b**).

Commented [LS1]: Primož, tele slike ne citiramo nikjer. Ne vem, če je tako OK. Že S11 ne omenjamo v glavnem članku.

Commented [PJ2R2]: Če nas noben recenzent ni na to opozoril se ne bi sekiral in bi obe slike kar pustil.

Table S2 shows changes in statistical values of Ir nanoparticles in TiO_xN_y–Ir sample before and after degradation. Less than 1 % of Ir nanoparticles are lost during EC-STAT. The average particle size, nearest neighbour distance, and circularity stay approximately the same. **Figure S10** shows the distributions for particle size, nearest neighbour distance, and circularity before and after EC-STAT.

Table S2: Changes in statistical values of Ir nanoparticles in TiO_xN_y–Ir sample before and after EC-STAT.

**TiO_xN_y–Ir SYSTEM STATISTICS**	**TiO_xN_y–Ir before EC-STAT**	**TiO_xN_y–Ir after EC-STAT**
Number of particles	1709	1698
Particle size (nm²)	1.53 ± 1.43	1.53 ± 1.38
Nearest neighbour distance (nm)	0.43 ± 0.23	0.44 ± 0.24

Circularity	0.84 ± 0.08	0.83 ± 0.09

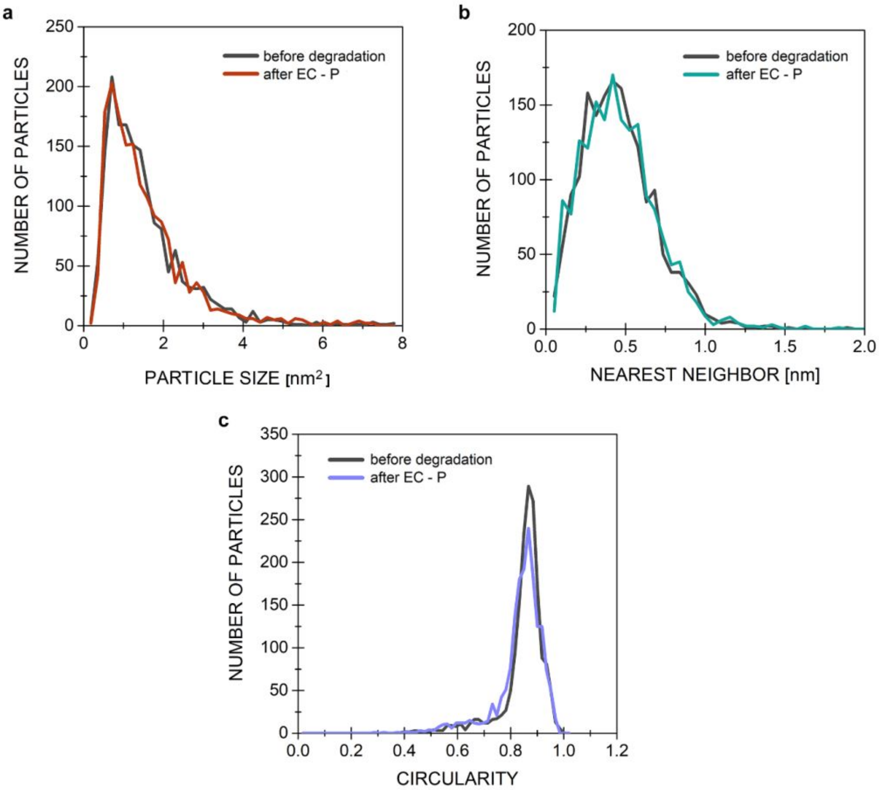

Figure S10: Particle size distribution (a), dispersion (b), and circularity (c) of Ir nanoparticles in TiO_xN_y–Ir sample before and after EC-STAT.

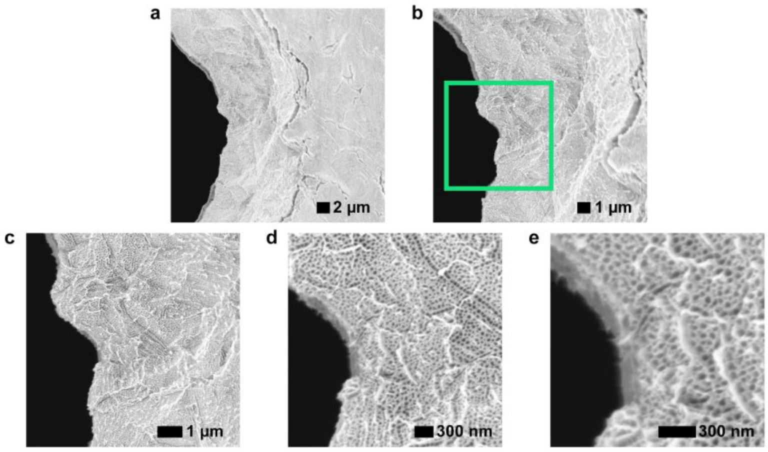

Figure S11: Scanning electron microscope images of TEM grid after anodic oxidation at different magnifications. Images **b, c, d, e** are magnified 2x, 4x, 10x and 20x relative to image **a**, respectively.

SI 8. TiO_xN_y electrochemistry

<u>Pre – treatment:</u> Initially, we focused on elucidation of TiO_xN_y electrochemistry. TiO_xN_y – based supports have raised much controversy over the recent years. Namely, initial studies claimed TiO_xN_y materials to be promising for OER [50,51]. However, later, more exact investigations by Behm's group refuted these claims by combining electrochemical measurements with online mass spectrometry analysis [52]. In their study, catalytic O_2 evolution was undoubtedly separated from intrinsic TiO_xN_y oxidation. For the latter case, it was shown that corresponding oxidation current onsets at approximately 1.2 V and reaches maximum at 1.7 V vs RHE. OER on TiO_xN_y electrode on the other hand proceeds only at potentials above 1.7 V [52]. Similar reasoning was followed in our study. Namely, in order to compare TiO_xN_y – based support synthesized in the present study to existing literature, initial electrochemical experiments were targeted at resolving TiO_xN_y – based grid's redox behaviour. For this purpose, cyclovoltammetric perturbation was employed. Distinct Faradaic current in the non-OER region is well resolved and should be ascribed to surface oxidation of nitride sites. This process shows irreversible redox behaviour in the selected potential window where current in consecutive potential cycles rapidly decreased **(Figure 5)**. Note that this is in close agreement with previously reported irreversible redox behaviour for TiO_xN_y [52]. The results from **Figure S12a** are of direct relevance for further electrochemical inspection of such supports. More specific, TiO_xN_y oxidation must be considered when OER catalysts are dispersed on TiO_xN_y support. In this case, the catalytic composite should undergo an electrochemical activation to ensure an adequate inhibition of TiO_xN_y Faradayic response at the onset of OER in order not to mistakenly ascribe TiO_xN_y electrochemical response to OER.

<u>OER:</u> Similar trends are obtained in the present study by comparing the herein investigated TiO_xN_y and preliminary TiO_xN_y–Ir analogues in a typical OER potential region. The current is significantly smaller in comparison to preliminary TiO_xN_y–Ir sample (**Figure S12b**). Therefore, TiO_xN_y can be considered essentially inactive for OER in the selected potential window.

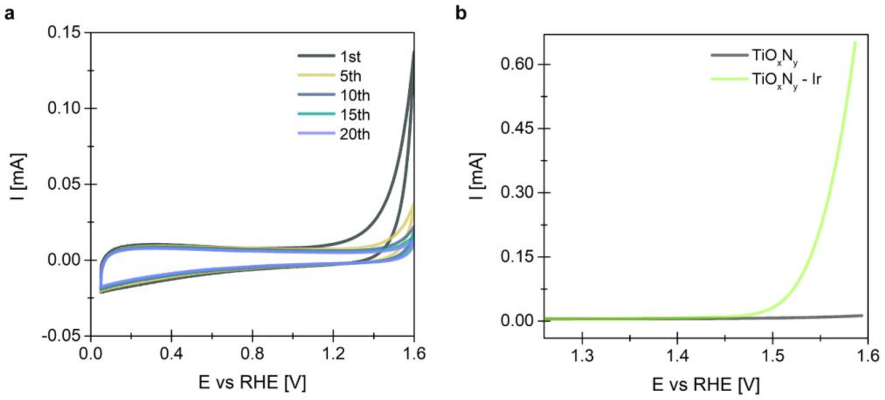

Figure S12: **a)** Electrochemical response of TiO_xN_y under cyclovoltammetric conditions (20 mV s^{-1}). **b)** Comparison of OER polarization curves (20 mV s^{-1}) for preliminary TiO_xN_y -Ir sample and TiO_xN_y.

References

(1) Hodnik, N.; Dehm, G.; Mayrhofer, K. J. J. Importance and Challenges of Electrochemical in Situ Liquid Cell Electron Microscopy for Energy Conversion Research. *Accounts of Chemical Research* **2016**, *49* (9), 2015–2022. https://doi.org/10.1021/acs.accounts.6b00330.

(2) Mayrhofer, K. J. J. J.; Meier, J. C.; Ashton, S. J.; Wiberg, G. K. H. H.; Kraus, F.; Hanzlik, M.; Arenz, M. Fuel Cell Catalyst Degradation on the Nanoscale. *Electrochemistry Communications* **2008**, *10*, 1144–1147. https://doi.org/10.1016/j.elecom.2008.05.032.

(3) Hodnik, N.; Cherevko, S. Spot the Difference at the Nanoscale: Identical Location Electron Microscopy in Electrocatalysis. *Current Opinion in Electrochemistry* **2019**, *15*, 73–82. https://doi.org/10.1016/J.COELEC.2019.03.007.

(4) Jiang, W.; Tang, F.; Gan, L. Electrochemical Stability of Au-TEM Grid with Carbon Supporting Film in Acid and Alkaline Electrolytes for Identical-Location TEM Study. *Journal of Electroanalytical Chemistry* **2018**, *826* (August), 46–51. https://doi.org/10.1016/j.jelechem.2018.08.026.

(5) Meier, J. C.; Katsounaros, I.; Galeano, C.; Bongard, H. J.; Topalov, A. a.; Kostka, A.; Karschin, A.; Schuth, F.; Mayrhofer, K. J. J.; Schüth, F.; Mayrhofer, K. J. J. Stability Investigations of Electrocatalysts on the Nanoscale. *Energy & Environmental Science* **2012**, *5*, 9319–9330. https://doi.org/10.1039/C2EE22550F.

(6) Schlögl, K.; Mayrhofer, K. J. J.; Hanzlik, M.; Arenz, M. Identical-Location TEM Investigations of Pt/C Electrocatalyst Degradation at Elevated Temperatures. *Journal of Electroanalytical Chemistry* **2011**, *662* (2), 355–360. https://doi.org/10.1016/j.jelechem.2011.09.003.

(7) Meier, J. C.; Galeano, C.; Katsounaros, I.; Topalov, A. a.; Kostka, A.; Schüth, F.; Mayrhofer, K. J. J. Degradation Mechanisms of Pt/C Fuel Cell Catalysts under Simulated Start–Stop Conditions. *ACS Catalysis* **2012**, *2*, 832–843. https://doi.org/10.1021/cs300024h.

(8) Perez-alonso, F. J.; Elkjær, C. F.; Shim, S. S.; Abrams, B. L.; Stephens, I. E. L.; Chorkendorff, I. Identical Locations Transmission Electron Microscopy Study of Pt / C Electrocatalyst Degradation during Oxygen Reduction Reaction. *Journal of Power Sources* **2011**, *196* (15), 6085–6091. https://doi.org/10.1016/j.jpowsour.2011.03.064.

(9) Zalitis, C. M.; Kramer, D.; Kucernak, A. R. Electrocatalytic Performance of Fuel Cell Reactions at Low Catalyst Loading and High Mass Transport. *Physical Chemistry Chemical Physics* **2013**, *15* (12), 4329–4340. https://doi.org/10.1039/c3cp44431g.

(10) Lin, X.; Zalitis, C. M.; Sharman, J.; Kucernak, A. Electrocatalyst Performance at the Gas/Electrolyte Interface under High-Mass-Transport Conditions: Optimization of the "Floating Electrode" Method. *ACS Applied Materials & Interfaces* **2020**. https://doi.org/10.1021/acsami.0c12718.

(11) Jackson, C.; Raymakers, L. F. J. M.; Mulder, M. J. J.; Kucernak, A. R. J. Assessing Electrocatalyst Hydrogen Activity and CO Tolerance: Comparison of Performance Obtained

Using the High Mass Transport 'Floating Electrode' Technique and in Electrochemical Hydrogen Pumps. *Applied Catalysis B: Environmental* **2020**, *268* (November 2019), 118734. https://doi.org/10.1016/j.apcatb.2020.118734.

(12) Zalitis, C.; Kucernak, A.; Lin, X.; Sharman, J. Electrochemical Measurement of Intrinsic Oxygen Reduction Reaction Activity at High Current Densities as a Function of Particle Size for Pt4- XCox/C (x = 0, 1, 3) Catalysts. *ACS Catalysis* **2020**, *10* (7), 4361–4376. https://doi.org/10.1021/acscatal.9b04750.

(13) Hrnjić, A.; Ruiz-Zepeda, F.; Gaberšček, M.; Bele, M.; Suhadolnik, L.; Hodnik, N.; Jovanovič, P. Modified Floating Electrode Apparatus for Advanced Characterization of Oxygen Reduction Reaction Electrocatalysts. *Journal of The Electrochemical Society* **2020**, *167* (16), 166501. https://doi.org/10.1149/1945-7111/abc9de.

(14) Hrnjic, A.; Kamšek, A. R.; Pavlišič, A.; Šala, M.; Bele, M.; Moriau, L.; Gatalo, M.; Ruiz-Zepeda, F.; Jovanovič, P.; Hodnik, N. Observing, Tracking and Analysing Electrochemically Induced Atomic-Scale Structural Changes of an Individual Pt-Co Nanoparticle as a Fuel Cell Electrocatalyst by Combining Modified Floating Electrode and Identical Location Electron Microscopy. *Electrochimica Acta* **2021**, 138513. https://doi.org/10.1016/j.electacta.2021.138513.

(15) Jovanovič, P.; Stojanovski, K.; Bele, M.; Dražić, G.; Koderman Podboršek, G.; Suhadolnik, L.; Gaberšček, M.; Hodnik, N. Methodology for Investigating Electrochemical Gas Evolution Reactions: Floating Electrode as a Means for Effective Gas Bubble Removal. *Analytical Chemistry* **2019**, *91* (16), 10353–10356. https://doi.org/10.1021/acs.analchem.9b01317.

(16) Kucernak, A. R.; Toyoda, E. Studying the Oxygen Reduction and Hydrogen Oxidation Reactions under Realistic Fuel Cell Conditions. *Electrochemistry Communications* **2008**, *10* (11), 1728–1731. https://doi.org/10.1016/j.elecom.2008.09.001.

(17) Moriau, L. J.; Hrnjić, A.; Pavlišič, A.; Kamšek, A. R.; Petek, U.; Ruiz-Zepeda, F.; Šala, M.; Pavko, L.; Šelih, V. S.; Bele, M.; Jovanovič, P.; Gatalo, M.; Hodnik, N. Resolving the Nanoparticles' Structure-Property Relationships at the Atomic Level: A Study of Pt-Based Electrocatalysts. *iScience* **2021**, *24* (2), 102102. https://doi.org/10.1016/j.isci.2021.102102.

(18) Lončar, A.; Escalera-López, D.; Ruiz-Zepeda, F.; Hrnjić, A.; Šala, M.; Jovanovič, P.; Bele, M.; Cherevko, S.; Hodnik, N. Sacrificial Cu Layer Mediated the Formation of an Active and Stable Supported Iridium Oxygen Evolution Reaction Electrocatalyst. *ACS Catalysis* **2021**, *11* (20), 12510–12519. https://doi.org/10.1021/acscatal.1c02968.

(19) Suhadolnik, L.; Bele, M.; Marinko, Ž. An Apparatus for Anodic Oxidation of Very Small Metal Grids. EP21163572.7, 2021.

(20) Podboršek, G. K.; Kamšek, A. R.; Lončar, A.; Bele, M.; Suhadolnik, L.; Jovanovič, P.; Hodnik, N. Atomically-Resolved Structural Changes of Ceramic Supported Nanoparticulate Oxygen Evolution Reaction Ir Catalyst. *Electrochimica Acta* **2022**, 140800. https://doi.org/10.1016/j.electacta.2022.140800.

(21) Lazaridis, T.; Stühmeier, B. M.; Gasteiger, H. A.; El-Sayed, H. A. Capabilities and Limitations of Rotating Disk Electrodes versus Membrane Electrode Assemblies in the Investigation of Electrocatalysts. *Nature Catalysis* **2022**, *5* (5), 363–373. https://doi.org/10.1038/s41929-022-00776-5.

(22) Zeradjanin, A. R. Frequent Pitfalls in the Characterization of Electrodes Designed for Electrochemical Energy Conversion and Storage. *ChemSusChem* **2018**, *11* (8), 1278–1284. https://doi.org/10.1002/cssc.201702287.

(23) El-Sayed, H. A.; Weiß, A.; Olbrich, L. F.; Putro, G. P.; Gasteiger, H. A. OER Catalyst Stability Investigation Using RDE Technique: A Stability Measure or an Artifact? *J Electrochem Soc* **2019**, *166* (8), F458–F464. https://doi.org/10.1149/2.0301908jes.

(24) Bele, M.; Jovanovič, P.; Marinko, Ž.; Drev, S.; Šelih, V. S.; Kovač, J.; Gaberšček, M.; Koderman Podboršek, G.; Dražić, G.; Hodnik, N.; Kokalj, A.; Suhadolnik, L. Increasing the Oxygen-Evolution Reaction Performance of Nanotubular Titanium Oxynitride-Supported Ir Nanoparticles by a Strong Metal–Support Interaction. *ACS Catalysis* **2020**, *10* (22), 13688–13700. https://doi.org/10.1021/acscatal.0c03688.

(25) Trasatti, S. Electrocatalysis in the Anodic Evolution of Oxygen and Chlorine. *Electrochimica Acta* **1984**, *29* (11), 1503–1512. https://doi.org/10.1016/0013-4686(84)85004-5.

(26) Da Silva, L. A.; Alves, V. A.; Da Silva, M. A. P.; Trasatti, S.; Boodts, J. F. C. Oxygen Evolution in Acid Solution on IrO2 + TiO2 Ceramic Films. A Study by Impedance, Voltammetry and SEM. *Electrochimica Acta* **1997**, *42* (2), 271–281. https://doi.org/10.1016/0013-4686(96)00160-0.

(27) Wei, C.; Sun, S.; Mandler, D.; Wang, X.; Qiao, S. Z.; Xu, Z. J. Approaches for Measuring the Surface Areas of Metal Oxide Electrocatalysts for Determining Their Intrinsic Electrocatalytic Activity. *Chemical Society Reviews* **2019**, *48* (9), 2518–2534. https://doi.org/10.1039/c8cs00848e.

(28) Watzele, S.; Hauenstein, P.; Liang, Y.; Xue, S.; Fichtner, J.; Garlyyev, B.; Scieszka, D.; Claudel, F.; Maillard, F.; Bandarenka, A. S. Determination of Electroactive Surface Area of Ni-, Co-, Fe-, and Ir-Based Oxide Electrocatalysts. *ACS Catalysis* **2019**, *9* (10), 9222–9230. https://doi.org/10.1021/acscatal.9b02006.

(29) Oh, H. S.; Nong, H. N.; Reier, T.; Bergmann, A.; Gliech, M.; Ferreira De Araújo, J.; Willinger, E.; Schlögl, R.; Teschner, D.; Strasser, P. Electrochemical Catalyst-Support Effects and Their Stabilizing Role for IrOxNanoparticle Catalysts during the Oxygen Evolution Reaction. *Journal of the American Chemical Society* **2016**, *138* (38), 12552–12563. https://doi.org/10.1021/jacs.6b07199.

(30) Marshall, A. T.; Haverkamp, R. G. Electrocatalytic Activity of IrO2-RuO2 Supported on Sb-Doped SnO2 Nanoparticles. *Electrochimica Acta* **2010**, *55* (6), 1978–1984. https://doi.org/10.1016/j.electacta.2009.11.018.

(31) Oh, H.-S.; Nong, H. N.; Reier, T.; Gliech, M.; Strasser, P. Oxide-Supported Ir Nanodendrites with High Activity and Durability for the Oxygen Evolution Reaction in Acid PEM Water Electrolyzers. *Chem. Sci.* **2015**, *6* (6), 3321–3328. https://doi.org/10.1039/C5SC00518C.

(32) Liu, G.; Xu, J.; Wang, Y.; Wang, X. An Oxygen Evolution Catalyst on an Antimony Doped Tin Oxide Nanowire Structured Support for Proton Exchange Membrane Liquid Water Electrolysis. *Journal of Materials Chemistry A* **2015**, *3* (41), 20791–20800. https://doi.org/10.1039/c5ta02942b.

(33) Moriau, L.; Bele, M.; Marinko, Ž.; Ruiz-Zepeda, F.; Koderman Podboršek, G.; Šala, M.; Šurca, A. K.; Kovač, J.; Arčon, I.; Jovanovič, P.; Hodnik, N.; Suhadolnik, L. Effect of the Morphology of the High-Surface-Area Support on the Performance of the Oxygen-Evolution Reaction for Iridium Nanoparticles. *ACS Catalysis* **2021**, *11* (2), 670–681. https://doi.org/10.1021/acscatal.0c04741.

(34) Moriau, L.; Koderman Podboršek, G.; Surca, A. K.; Semsari Parpari, S.; Šala, M.; Petek, U.; Bele, M.; Jovanovič, P.; Genorio, B.; Hodnik, N. Enhancing Iridium Nanoparticles' Oxygen Evolution Reaction Activity and Stability by Adjusting the Coverage of Titanium Oxynitride Flakes on Reduced Graphene Oxide Nanoribbons' Support. *Adv Mater Interfaces* **2021**, *8* (17), 2100900. https://doi.org/10.1002/admi.202100900.

(35) Pickup, P. G.; Birss, V. I. A Model for Anodic Hydrous Oxide Growth at Iridium. *Journal of Electroanalytical Chemistry* **1987**, *220* (1), 83–100. https://doi.org/10.1016/0022-0728(87)88006-3.

(36) Michell, D.; Rand, D. A. J.; Woods, R. Analysis of the Anodic Oxygen Layer on Iridium by X-Ray Emission, Electron Diffraction and Electron Microscopy. *Journal of Electroanalytical Chemistry and Interfacial Electrochemistry* **1977**, *84* (1), 117–126. https://doi.org/10.1016/S0022-0728(77)80234-9.

(37) Otten, J. M.; Visscher, W. The Anodic Behaviour of Iridium. *Journal of Electroanalytical Chemistry and Interfacial Electrochemistry* **1974**, *55* (1), 13–21. https://doi.org/10.1016/S0022-0728(74)80468-7.

(38) Rand, D. A. J.; Woods, R. Cyclic Voltammetric Studies on Iridium Electrodes in Sulphuric Acid Solutions. *Journal of Electroanalytical Chemistry and Interfacial Electrochemistry* **1974**, *55* (3), 375–381. https://doi.org/10.1016/S0022-0728(74)80431-6.

(39) Schindelin, J.; Arganda-Carreras, I.; Frise, E.; Kaynig, V.; Longair, M.; Pietzsch, T.; Preibisch, S.; Rueden, C.; Saalfeld, S.; Schmid, B.; Tinevez, J. Y.; White, D. J.; Hartenstein, V.; Eliceiri, K.; Tomancak, P.; Cardona, A. Fiji: An Open-Source Platform for Biological-Image Analysis. *Nature Methods* **2012**, *9* (7), 676–682. https://doi.org/10.1038/nmeth.2019.

(40) Hrnjić, A.; Ruiz-Zepeda, F.; Gaberšček, M.; Bele, M.; Suhadolnik, L.; Hodnik, N.; Jovanovič, P. Modified Floating Electrode Apparatus for Advanced Characterization of Oxygen Reduction Reaction Electrocatalysts. *Journal of The Electrochemical Society* **2020**, *167* (16), 166501. https://doi.org/10.1149/1945-7111/abc9de.

(41) Trasatti, S. Electrocatalysis in the Anodic Evolution of Oxygen and Chlorine. *Electrochimica Acta* **1984**, *29* (11), 1503–1512. https://doi.org/10.1016/0013-4686(84)85004-5.

(42) Da Silva, L. A.; Alves, V. A.; Da Silva, M. A. P.; Trasatti, S.; Boodts, J. F. C. Oxygen Evolution in Acid Solution on IrO2 + TiO2 Ceramic Films. A Study by Impedance, Voltammetry and SEM. *Electrochimica Acta* **1997**, *42* (2), 271–281. https://doi.org/10.1016/0013-4686(96)00160-0.

(43) Wei, C.; Sun, S.; Mandler, D.; Wang, X.; Qiao, S. Z.; Xu, Z. J. Approaches for Measuring the Surface Areas of Metal Oxide Electrocatalysts for Determining Their Intrinsic Electrocatalytic Activity. *Chemical Society Reviews* **2019**, *48* (9), 2518–2534. https://doi.org/10.1039/c8cs00848e.

(44) Han, J. H.; Bang, J. H. A Hollow Titanium Oxynitride Nanorod Array as an Electrode Substrate Prepared by the Hot Ammonia-Induced Kirkendall Effect. *Journal of Materials Chemistry A* **2014**, *2* (27), 10568–10576. https://doi.org/10.1039/c4ta01469c.

(45) Pfeifer, V.; Jones, T. E.; Velasco Vélez, J. J.; Massué, C.; Arrigo, R.; Teschner, D.; Girgsdies, F.; Scherzer, M.; Greiner, M. T.; Allan, J.; Hashagen, M.; Weinberg, G.; Piccinin, S.; Hävecker, M.; Knop-Gericke, A.; Schlögl, R. The Electronic Structure of Iridium and Its Oxides. *Surface and Interface Analysis* **2016**, *48* (5), 261–273. https://doi.org/10.1002/sia.5895.

(46) Ohsaka, T.; Izumi, F.; Fujiki, Y. Raman Spectrum of Anatase, TiO2, MgF2, ZnF2, FeF2, and MnF2. *Journal of Raman Spectroscopy* **1978**, *7* (6), 321–324. https://doi.org/10.1002/jrs.1250070606.

(47) Porto, S. P. S.; Fleury, P. A.; Damen, T. C. Raman Spectra of TiO2. *Physical Review* **1967**, *154* (2), 522. https://doi.org/10.1103/PhysRev.154.522.

(48) Ding, Z. H.; Yao, B.; Qiu, L. X.; Lv, T. Q. Raman Scattering Investigation of Nanocrystalline δ-TiNx Synthesized by Solid-State Reaction. *Journal of Alloys and Compounds* **2006**, *421* (1–2), 247–251. https://doi.org/10.1016/j.jallcom.2005.11.017.

(49) Spengler, W.; Kaiser, R.; Christensen, A. N.; Müller-Vogt, G. Raman Scattering, Superconductivity, and Phonon Density of States of Stoichiometric and Nonstoichiometric TiN. *Physical Review B* **1978**, *17* (3), 1095–1101. https://doi.org/10.1103/PhysRevB.17.1095.

(50) Azuma, M.; Nakato, Y.; Tsubomura, H. Oxygen and Chlorine Evolution on Niobium-, Zirconium- and Other Metal-Nitride Amorphous Thin Film Electrodes Prepared by the Reactive RF Sputtering Technique. *Journal of Electroanalytical Chemistry* **1988**, *255* (1–2), 179–198. https://doi.org/10.1016/0022-0728(88)80013-5.

(51) Wang, W.; Savadogo, O.; Ma, Z.-F. Preparation of New Titanium Oxy Nitride Based Electro Catalysts Using an Anhydrous Sol-Gel Method for Water Electrolysis in Acid Medium. *International Journal of Hydrogen Energy* **2012**, *37* (9), 7405–7417. https://doi.org/10.1016/J.IJHYDENE.2012.02.025.

(52) Gebauer, C.; Fischer, P.; Wassner, M.; Diemant, T.; Jusys, Z.; Hüsing, N.; Behm, R. J. J. Performance of Titanium Oxynitrides in the Electrocatalytic Oxygen Evolution Reaction. *Nano Energy* **2016**, *29*, 136–148. https://doi.org/10.1016/j.nanoen.2016.05.034.
